# Crystal structure of di-μ-chloro­acetato-hexa­kis­(di­methyl­formamide)­tetra­kis­(μ-*N*,2-dioxido­benzene-1-carboximidato)tetra­manganese(III)disodium dimethyl­formamide disolvate

**DOI:** 10.1107/S1600536814024441

**Published:** 2014-11-15

**Authors:** Connor I. Daly, Matthias Zeller, Curtis M. Zaleski

**Affiliations:** a Department of Chemistry, Shippensburg University, 1871 Old Main Dr., Shippensburg, PA 17257, USA; b Department of Chemistry, Youngstown State University, 1 University Plaza, Youngstown, OH 44555, USA

**Keywords:** manganese, metallacrown, coordination compound, crystal structure

## Abstract

The synthesis, crystal structure, and FT–IR data for the title compound, [Na_2_Mn_4_(C_2_H_2_ClO_2_)_2_(C_7_H_4_NO_3_)_4_(C_3_H_7_NO)_6_]·2C_3_H_7_NO or Na_2_(O_2_CCH_2_Cl)_2_[12-MC_Mn^III^_ _N(shi)_-4](DMF)_6_·2DMF, where MC is metallacrown, shi^3−^ is salicyl­hydroximate, and DMF is *N*,*N*-di­methyl­formamide, is reported. The macrocyclic metallacrown consists of an –[Mn^III^—N—O]_4_– ring repeat unit and the metallacrown captures two Na^+^ ions in the central cavity above and below the plane of the metallacrown. Each Na^+^ ion is seven-coordinate and is bridged to two ring Mn^III^ ions, through either a coordinating DMF mol­ecule or a chloro­acetate anion. The ring Mn^III^ ions have either a tetra­gonally distorted octa­hedral geometry or a distorted square-pyramidal geometry. Weak C—H⋯O inter­actions, in addition to pure van der Waals forces, contribute to the overall packing of the mol­ecules. The complete molecule has inversion symmetry and is disordered over two sets of sites with an occupancy ratio of 0.8783 (7):0.1217 (7). The solvent molecule is also disordered over two sets of sites, with an occupancy ratio of 0.615 (5):0.385 (5).

## Abstract 2

The title compound consists of a macrocyclic ring with an Mn^III^—N—O repeat unit that occurs four times, producing a mol­ecule with an overall square structure. Two Na^+^ ions are captured above and below the central cavity of the mol­ecule.

## Chemical context   

Metallacrowns (MCs) are a family of macrocyclic inorganic complexes with structural and functional similarity to crown ethers (Mezei *et al.*, 2007 ▶). As crown ethers are composed of a –[C—C—O]_*n*_– repeat unit, metallacrowns possess an –[M—N—O]_*n*_– repeat unit. While metallacrowns can selectively bind alkali metal ions in the central cavity similar to crown ethers, MCs have also found applications as single-mol­ecule magnets, anti­microbial agents, and building blocks for one-, two-, and three-dimensional solids (Mezei *et al.*, 2007 ▶). The controllable synthesis of macrocyclic inorganic mol­ecules is of importance if the properties of a mol­ecule are to be tailored for a specific application. However, inorganic reactions can be unpredictable due to labile metal–ligand coordination bonds. In addition, the products of many inorganic reactions can be serendipitous in nature (Saalfrank *et al.*, 2008 ▶). Thus, the ability to controllably substitute components of a mol­ecular class allow for the fine-tuning of mol­ecular properties.

The 12-MC_Mn^III^_ _N(shi)_-4 class of mol­ecules, with Mn^III^ ions as the ring metal and salicyl­hydroximate (shi^3−^) ligands composing the MC framework, provide a rich opportunity to perform substitution reactions. These metallacrowns can bind a variety of metal ions in the central cavity such as Mn^II^, Li^+^, Na^+^, K^+^, Ca^2+^, and Ln^III^ ions (*Ln* is a lanthanide) (Lah & Pecoraro, 1989 ▶, 1991 ▶; Gibney *et al.*, 1996 ▶; Kessissoglou *et al.*, 2002 ▶; Koumousi *et al.*, 2011 ▶; Azar *et al.*, 2014 ▶). Also, while the MC framework is neutral due to the four Mn^III^ ions and four shi^3−^ ligands, the addition of the central metal ion necessitates counter-anions, which also provide another substitution point. Thus, the 12-MC_Mn^III^_ _N(shi)_-4 structure affords an opportunity to investigate the substitution capability of MCs. 

Herein we report the synthesis and crystal structure of Na_2_(O_2_CCH_2_Cl)_2_[12-MC_Mn^III^_ _N(shi)_-4](DMF)_6_·2DMF (DMF is *N*,*N*-dimethylformamide). This metalla­crown demon­strates the inclusion of chloro­acetate into the 12-MC_Mn^III^_ _N(shi)_-4 structure, which serves as a bridging anion between ring Mn^III^ ions and Na^+^ captured above and below the central MC cavity.
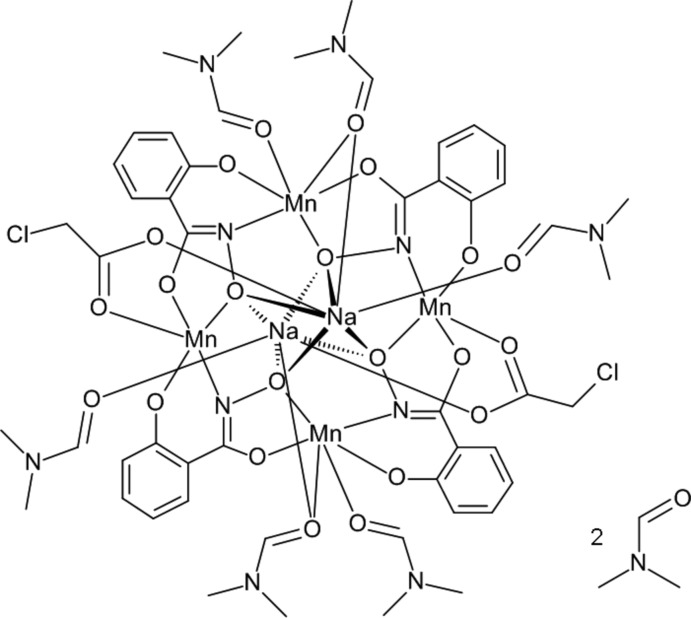


## Structural commentary   

**Figure 1:**
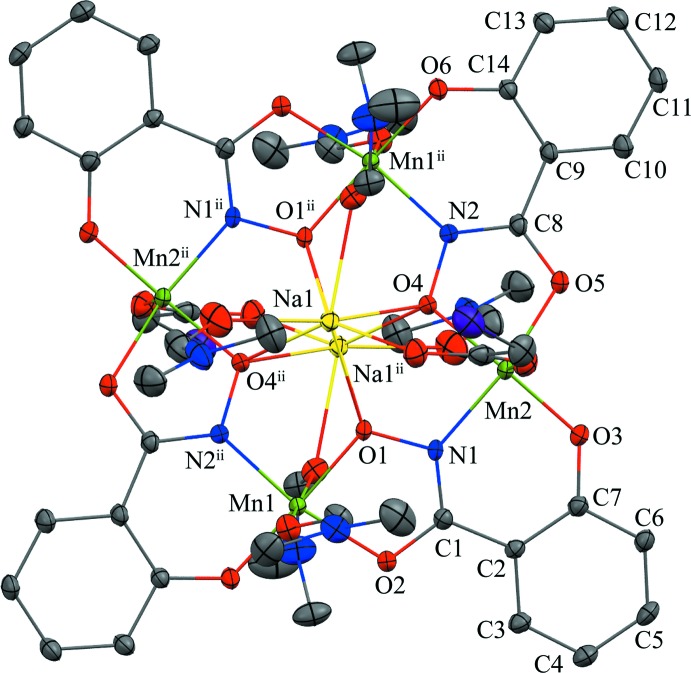
Molecular structure of Na_2_(O_2_CCH_2_Cl)_2_[12-MC_Mn^III^_ _N(shi)_-4](DMF)_6_·2DMF (top view). The displacement ellipsoid plot is at the 50% probability level. Atom labels for all non-H atoms on one asymmetric unit of the 12-MC-4 framework and selected symmetry-equivalent atoms have been provided. For clarity, atom labels for the axial DMF and chloro­acetate ligands have been omitted; those labels may be found in Fig. 2 ▶. H atoms and the lattice solvent mol­ecules have been omitted for clarity. Color scheme: green Mn^III^, yellow Na^+^, purple chlorine, red oxygen, blue nitro­gen, and gray carbon. [Symmetry code: (ii) −*x* + 1, −*y*, −*z* + 1.]

**Figure 2:**
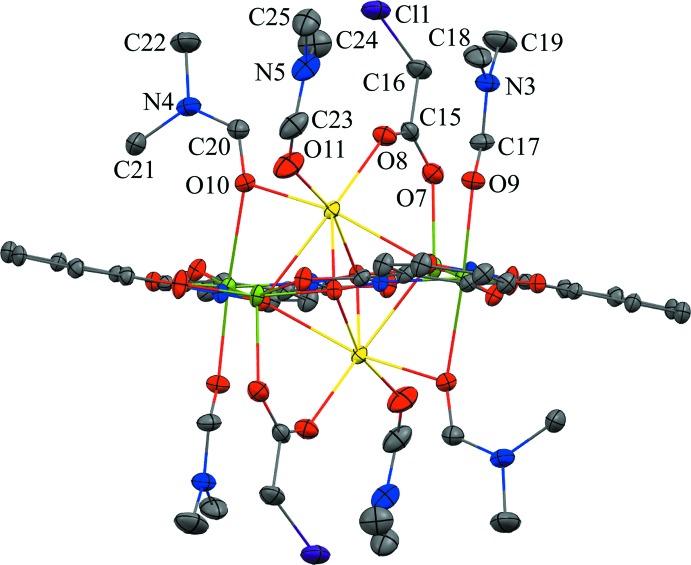
Molecular structure of Na_2_(O_2_CCH_2_Cl)_2_[12-MC_Mn^III^_ _N(shi)_-4](DMF)_6_·2DMF (side view). The stepped or ruffled character of the structure is emphasised in this view. Atom labels for all non-hydrogen atoms of the axial DMF and chloro­acetate ligands on one asymmetric unit have been provided. See Fig. 1 ▶ for display details.

The title compound consists of the typical 12-MC_Mn^III^_ _N(shi)_-4 framework with four Mn^III^—N—O repeating units producing an overall square-geometry mol­ecule (Fig. 1 ▶). As in other di-sodium 12-MC_Mn^III^_ _N(shi)_-4 complexes (Lah & Pecoraro, 1991 ▶; Gibney *et al.*, 1996 ▶; Kessissoglou *et al.*, 2002 ▶; Azar *et al.*, 2014 ▶), an inversion center is located in the central MC cavity produced by the oxime oxygen atoms of the shi^3−^ ligands. In addition, two Na^+^ ions are captured in the central cavity on opposite faces of the MC (Fig. 2 ▶). A chloro­acetate anion bridges each Na^+^ ion to a ring manganese ion. The entire mol­ecule (metallacrown, chloro­acetate counter-anions, and coordinating DMF mol­ecules) is disordered over two sites with an occupancy ratio of 0.8783 (7):0.1217 (7) (complete refinement details are given below); thus, a description will only be given for the higher occupancy component. The metallacrown is nearly planar, but it can be considered to possess a stepped structure, *i.e.* the MC is ruffled (Fig. 2 ▶). Charge neutrality is maintained for the mol­ecule by the presence of four Mn^III^ and two Na^+^ cations and four shi^3−^ and two chloro­acetate anions. The oxidation state assignment of the ring Mn^III^ ions is supported by the average bond lengths, bond-valence-sum (BVS) calculations, and the presence of elongated axial bond lengths expected for a high-spin *d* ^4^ electron configuration (Liu & Thorp, 1993 ▶). For Mn1, the average bond length is 2.05 Å and the BVS value is 3.06 valence units (v.u.), and for Mn2 the average bond length is 1.96 Å and the BVS value is 2.98  v.u.

The coordination geometry about Mn1 is best described as a tetra­gonally distorted octa­hedron with the equatorial ligands comprised of an oxime nitro­gen atom and a phenolate oxygen atom from one shi^3−^ ligand and an oxime oxygen atom and carbonyl oxygen atom from a second shi^3−^ ligand. The Jahn–Teller axis is completed by the carbonyl oxygen atoms of two *trans* DMF mol­ecules (average Mn—O_JT_ = 2.31 Å). The carbonyl oxygen atom (O10) of one of the DMF mol­ecules also serves as a one-atom bridge to the central Na^+^ ion. For Mn2, the coordination geometry is best described as distorted square-pyramidal with a τ value of 0.05, where τ = 0 for ideal square-pyramidal geometry and τ = 1 for ideal trigonal-bipyramidal geometry (Addison *et al.*, 1984 ▶). The basal ligands are comprised of an oxime nitro­gen atom and a phenolate oxygen atom from one shi^3−^ ligand and an oxime oxygen atom and a carbonyl oxygen atom from a second shi^3−^ ligand. The oxygen atom of a chloro­acetate anion binds in the elongated apical direction [Mn2—O7: 2.1202 (15) Å]. The chloro­acetate forms a three-atom bridge to the central Na^+^ ion. Each Na^+^ ion is seven coordinate. The four oxime oxygen atoms of the MC cavity form a square face below the Na^+^ ion, and three oxygen atoms form a triangular face above the ion. The three oxygen atoms are from the bridging chloro­acetate anion, a carbonyl oxygen atom of the bridging DMF mol­ecule, and a carbonyl oxygen atom of a terminal DMF mol­ecule. Lastly two DMF mol­ecules, which are related by the inversion center at (0.5, 0.0, 0.5), are located in the lattice and are disordered over two sites with different orientations with an occupancy ratio of 0.615 (5):0.385 (5).

## Supra­molecular features   

**Figure 3:**
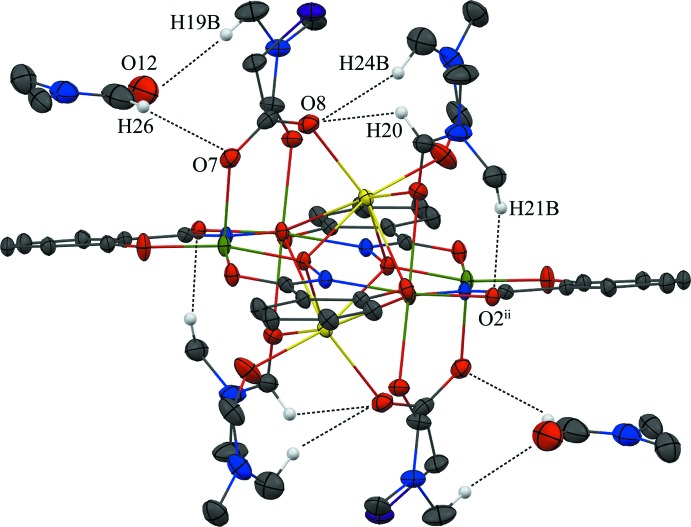
Intra- and inter­molecular hydrogen bonding within the metallacrown itself and between the MC and the lattice DMF mol­ecule. For clarity, only the H atoms (white) involved in the hydrogen bonding have been included and only the atoms involved in the hydrogen bonding have been labelled. See Fig. 1 ▶ for display details. [Symmetry code: (ii) −*x* + 1, −*y*, −*z* + 1.]

No strong directional inter­molecular inter­actions are observed between the Na_2_(O_2_CCH_2_Cl)_2_[12-MC_Mn^III^_ _N(shi)_-4](DMF)_6_ mol­ecules, but a number of weak intra­molecular and inter­molecular C—H⋯O inter­actions exist (Table 1 ▶). The intra­molecular inter­actions exist between an oxygen atom of the bridging chloro­acetate anion and a methyl carbon atom of a coordinating DMF mol­ecule and a carbonyl carbon atom of another coordinating DMF mol­ecule, and between the carbonyl oxygen atom of a shi^3−^ ligand and the methyl carbon atom of a coordinating DMF mol­ecule (Fig. 3 ▶). The inter­molecular inter­actions exist between the carbonyl oxygen atom of a lattice DMF mol­ecule and the methyl carbon atoms of two different coordinating DMF mol­ecules, between an oxygen atom of a chloro­acetate and a carbonyl carbon atom of a lattice DMF mol­ecule, between a carbonyl oxygen atom of a coordinating DMF mol­ecule and a methyl carbon atom of a coordinating DMF mol­ecule of an adjacent MC, and between a carbonyl oxygen atom of a shi^3−^ ligand and the methyl carbon atom of a coordinating DMF mol­ecule of a neighboring MC (Figs. 3 ▶ and 4 ▶). These weak C—H⋯O inter­actions, in addition to pure van der Waals forces, contribute to the overall packing of the mol­ecules.

## Database survey   

The X-ray crystal structures of four other di-sodium 12-MC_Mn^III^_ _N(shi)_-4 complexes have been reported: Na_2_Cl_2_[12-MC_Mn^III^_ _N(shi)_-4](DMF)_6_·3DMF (Lah & Pecoraro, 1991 ▶), Na_2_Br_2_[12-MC_Mn^III^_ _N(shi)_-4](DMF)_8_ (Gibney *et al.*, 1996 ▶), Na_2_(NCS)_2_[12-MC_Mn^III^_ _N(shi)_-4](DMF)_8_, (Kessissoglou *et al.*, 2002 ▶) and Na_2_(O_2_CCH_3_)_2_[12-MC_Mn^III^_ _N(shi)_-4](DMF)_6_·2DMF·1.60H_2_O (Azar *et al.*, 2014 ▶). As in the other four structures, the title compound has a ruffled structure and the Na^+^ ions bind on opposite faces of the MC. In the chloride, bromide, acetate, and chloro­acetate versions, the anion bridges between a ring Mn^III^ ion and the central Na^+^ ion. However, in the thio­cyanate analogue, the anion does not bridge between the ring Mn^III^ ions and the central Na^+^ ions. Comparing the two carboxyl­ate anion structures, the metallacrown cavity radius of each 12-MC_Mn^III^_ _N(shi)_-4 is similar with 0.55 Å for the acetate version and 0.56 Å for the chloro­acetate analogue. However, the Na^+^ ions in the chloro­acetate MC more closely approach the mean plane produced by the manganese(III) ions (Mn^III^MP) and the mean plane produced by the oxime oxygen atoms (O_ox_MP). For the acetate version, the Na^+^ ion to Mn^III^MP distance is approximately 1.65 Å, and the Na^+^ ion to the O_ox_MP distance is 1.66 Å. For the chloro­acetate version, the Na^+^–Mn^III^MP distance is 1.62 Å, and the Na^+^–O_ox_MP distance is 1.63 Å. Since the Na^+^ ions of the chloro­acetate version more closely approach the MC, the Na^+^–-Na^+^ distance [3.254 (4) Å] is slightly smaller than that observed for the acetate version [3.3364 (9) Å].

## Synthesis and crystallization   

The title compound was synthesized by first dissolving manganese(II) acetate tetra­hydrate (2 mmol) in 4 ml of methanol and 4 ml of DMF, which resulted in a dark-orange solution. Then a mixture of salicyl­hydroxamic acid (2 mmol) and sodium chloro­acetate (2 mmol) in 5 ml of methanol and 5 ml of DMF was added to the manganese(II) acetate solution. The resulting dark-brown solution was stirred overnight and filtered the next day without the recovery of a precipitate. After slow evaporation of the dark-brown filtrate for 7 days, black, block-like crystals suitable for X-ray diffraction were recovered. The percent yield was 41% based on mang­anese(II) acetate tetra­hydrate. Elemental analysis for C_56_H_76_Cl_2_Mn_4_N_12_Na_2_O_24_ [FW = 1637.92 g mol^−1^] found % (calculated): C 40.68 (41.06); H 4.58 (4.68); N 9.98 (10.27). FT–IR bands (KBr pellet, cm^−1^): 1650, 1598, 1567, 1517, 1469, 1434, 1389, 1315, 1256, 1156, 1098, 1035, 935, 862, 771, 757, 687, 649, 611, 583, 477.

## Refinement   

Crystal data, data collection and structure refinement details are summarized in Table 2 ▶. The metallacrown mol­ecule, coordinating DMF mol­ecules, and chloro­acetate anion show whole-mol­ecule disorder over two sets of sites. The geometries of the two metallacrowns, coordinating DMF mol­ecules, and the coordinating chloro­acetate anions were restrained to be similar to each other (SAME command in *SHELXL*, s.u. = 0.02 Å). For the benzene ring carbon atoms (C2–C7, C9–C14 and C2*B*–C7*B*, C9*B*–C14*B*), oxime oxygen atom (O4 and O4*B*), and oxime nitro­gen atoms (N1, N2 and N1*B*, N2*B*) of the salicyl­hydroximate ligands, equivalent atoms were constrained to have pairwise identical anisotropic displacement parameters (ADPs). The ADPs of the sodium ions (Na1 and Na1*B*) were also constrained to be identical. For the coordinating DMF mol­ecules, the nitro­gen atoms (N3 and N3*B*, N4 and N4*B*, and N5 and N5*B*) have nearly the same atom positions, leading to highly correlated thermal parameters. To avoid correlation of the thermal parameters, the ADPs of equivalent nitro­gen atoms in the DMF mol­ecules were constrained to be identical. In addition, carbon, oxygen, and chlorine atoms of the chloro­acetate and carbon, oxygen, and nitro­gen atoms of the coordinating DMF mol­ecules were restrained to have similar *U*_*ij*_ components of the ADPs (s.u. = 0.04 Å^2^; SIMU restraint in *SHELXL*). Anisotropic displacement parameters of all atoms in the minor moiety of the coordinating DMF mol­ecule associated with N5*B* were restrained using an enhanced rigid-bond restraint for the 1,2- and 1,3 distances [RIGU command in *SHELXL*, s.u. = 0.004 Å^2^ for both 1,2- and 1,3 distances (Thorn *et al.*, 2012 ▶)]. Additionally, the following sodium–oxygen bond lengths were restrained to be similar (s.u. 0.02 Å): Na1—O1 and Na1*B*—O1*B*, Na1—O4 and Na1*B*—O4*B*, Na1—O8 and Na1*B*—O8*B*, and Na1—O11 and Na1*B*—O11*B*. Subject to these conditions, the occupancy ratio of the disordered metallacrown and associated anion and solvent mol­ecules refined to 0.8783 (7):0.1217 (7).

A lattice DMF mol­ecule, associated with N6, is disordered over two sets of sites with different orientations. The geometries of the two DMF mol­ecules were restrained to be similar to each other (SAME command in *SHELXL*, s.u. = 0.02 Å). The nitro­gen atoms (N6 and N6*B*) have nearly the same atom positions, leading to highly correlated displacement parameters. To avoid correlation of the displacement parameters, the ADPs of equivalent atoms were constrained to be identical. In addition, carbon, oxygen, and nitro­gen atoms of the DMF mol­ecule were restrained to have similar *U*_*ij*_ components of the ADPs (s.u. = 0.04 Å^2^; SIMU restraint in *SHELXL*). Subject to these restraints, the occupancy ratio of the disordered DMF mol­ecule refined to 0.615 (5):0.385 (5).

All hydrogen atoms were placed in calculated positions and refined as riding on their carrier atoms with C—H distances of 0.95 Å for *sp* ^2^ carbon atoms and 0.98 Å for methyl carbon atoms. The *U*_iso_ values for hydrogen atoms were set to a multiple of the value of the carrying carbon atom (1.2 times for *sp* ^2^-hybridized carbon atoms or 1.5 times for methyl carbon atoms and water oxygen atoms). Major disorder component methyl H atoms were allowed to rotate, but not to tip (AFIX 137 command in *SHELXL*). For the minor disorder component, methyl H atoms, the C—N—C—H torsion angles were constrained, as implemented in the AFIX 33 command in *SHELXL*.

## Supplementary Material

Crystal structure: contains datablock(s) I. DOI: 10.1107/S1600536814024441/pk2534sup1.cif

Structure factors: contains datablock(s) I. DOI: 10.1107/S1600536814024441/pk2534Isup2.hkl

CCDC reference: 1033085

Additional supporting information:  crystallographic information; 3D view; checkCIF report

## Figures and Tables

**Figure 4:**
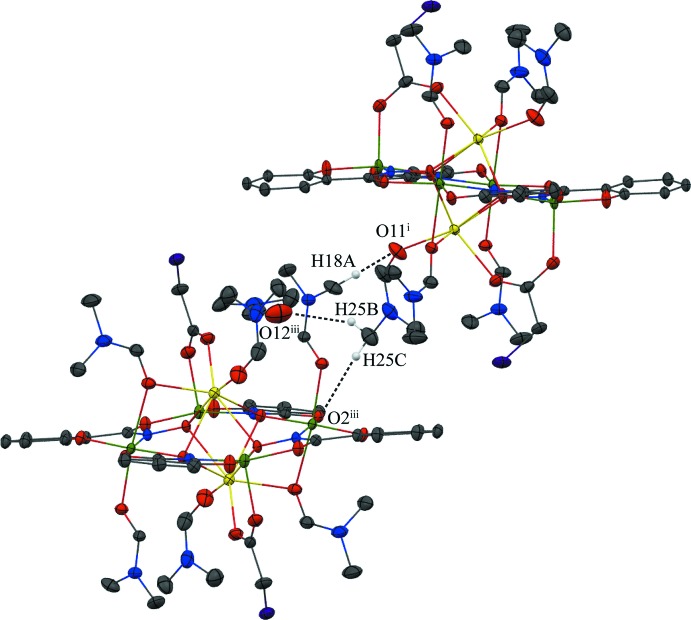
Inter­molecular hydrogen bonding between adjacent metallacrowns and between the MC and the lattice DMF mol­ecule. For clarity, only the H atoms (white) involved in the hydrogen bonding have been included and only the atoms involved in the hydrogen bonding have been labelled. See Fig. 1 ▶ for display details. [Symmetry codes: (i) −*x* + 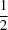, *y* + 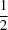, −*z* + 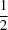; (iii) −*x* + 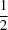, *y* − 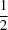, −*z* + 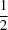.]

**Table 1:** Hydrogen-bond geometry (, )

*D*H*A*	*D*H	H*A*	*D* *A*	*D*H*A*
C18H18*A*O11^i^	0.98	2.63	3.516(5)	151
C19H19*B*O12	0.98	2.33	3.201(5)	148
C20H20O8	0.95	2.64	3.279(3)	125
C21H21*B*O2^ii^	0.98	2.48	3.433(3)	165
C24H24*B*O8	0.98	2.53	3.459(4)	158
C25H25*B*O12^iii^	0.98	2.55	3.309(6)	134
C25H25*C*O2^iii^	0.98	2.50	3.423(5)	157
C26H26O7	0.95	2.49	3.339(10)	149

Symmetry codes: (i) 
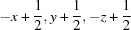
; (ii) 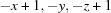; (iii) 
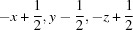
.

**Table 2:** Experimental details

Crystal data	
Chemical formula	[Na_2_Mn_4_(C_2_H_2_ClO_2_)_2_(C_7_H_4_NO_3_)_4_(C_3_H_7_NO)_6_]2C_3_H_7_NO
*M*_r_	1637.92
Crystal system, space group	Monoclinic, *P*2_1_/*n*
Temperature (K)	100
*a*, *b*, *c* ()	14.4457(7), 14.7091(6), 16.5663(8)
()	101.8584(17)
*V* (^3^)	3444.9(3)
*Z*	2
Radiation type	Mo *K*
(mm^1^)	0.89
Crystal size (mm)	0.32 0.30 0.21

Data collection	
Diffractometer	Bruker AXS D8 Quest CMOS
Absorption correction	Multi-scan (*SADABS*; Bruker, 2014 ▶)
*T*_min_, *T*_max_	0.645, 0.746
No. of measured, independent and observed [*I* > 2(*I*)] reflections	39712, 12392, 9900
*R*_int_	0.030
(sin /)_max_ (^1^)	0.757

Refinement	
*R*[*F* ^2^ > 2(*F* ^2^)], *wR*(*F* ^2^), *S*	0.046, 0.102, 1.06
No. of reflections	12392
No. of parameters	791
No. of restraints	748
H-atom treatment	H-atom parameters constrained
_max_, _min_ (e ^3^)	0.85, 0.45

Computer programs: *APEX2* and *SAINT* (Bruker, 2014 ▶), *SHELXS97* and *SHELXL2013* (Sheldrick, 2008 ▶), *SHELXLE* (Hbschle *et al.*, 2011 ▶), *Mercury* (Macrae *et al.*, 2006 ▶) and *publCIF* (Westrip, 2010 ▶).

## supplementary crystallographic information

### Crystal data

**Table:**

[Na_2_Mn_4_(C_2_H_2_ClO_2_)_2_(C_7_H_4_NO_3_)_4_(C_3_H_7_NO)_6_]·2C_3_H_7_NO	*F*(000) = 1688
*M**_r_* = 1637.92	*D*_x_ = 1.579 Mg m^−^^3^
Monoclinic, *P*2_1_/*n*	Mo *K*α radiation, λ = 0.71073 Å
*a* = 14.4457 (7) Å	Cell parameters from 9986 reflections
*b* = 14.7091 (6) Å	θ = 2.5–32.6°
*c* = 16.5663 (8) Å	µ = 0.89 mm^−^^1^
β = 101.8584 (17)°	*T* = 100 K
*V* = 3444.9 (3) Å^3^	Block, black
*Z* = 2	0.32 × 0.30 × 0.21 mm

### Data collection

**Table:**

Bruker AXS D8 Quest CMOS diffractometer	12392 independent reflections
Radiation source: I-mu-S microsource X-ray tube	9900 reflections with *I* > 2σ(*I*)
Laterally graded multilayer (Goebel) mirror monochromator	*R*_int_ = 0.030
ω and φ scans	θ_max_ = 32.6°, θ_min_ = 2.2°
Absorption correction: multi-scan (*SADABS*; Bruker, 2014)	*h* = −18→21
*T*_min_ = 0.645, *T*_max_ = 0.746	*k* = −16→22
39712 measured reflections	*l* = −25→24

### Refinement

**Table:**

Refinement on *F*^2^	Primary atom site location: structure-invariant direct methods
Least-squares matrix: full	Secondary atom site location: difference Fourier map
*R*[*F*^2^ > 2σ(*F*^2^)] = 0.046	Hydrogen site location: inferred from neighbouring sites
*wR*(*F*^2^) = 0.102	H-atom parameters constrained
*S* = 1.06	*w* = 1/[σ^2^(*F*_o_^2^) + (0.0286*P*)^2^ + 3.6909*P*] where *P* = (*F*_o_^2^ + 2*F*_c_^2^)/3
12392 reflections	(Δ/σ)_max_ = 0.001
791 parameters	Δρ_max_ = 0.85 e Å^−^^3^
748 restraints	Δρ_min_ = −0.45 e Å^−^^3^

### Special details

**Table:**

Experimental. The metallacrown molecule, coordinating DMF molecules, and chloroacetate anion show whole molecule disorder over two sites. The geometries of the two metallacrowns, coordinating DMF molecules, and the coordinating chloroacetate anions were restrained to be similar to each other (SAME command in *SHELXL*, e.s.d. = 0.02 Angstrom). For the benzene ring carbon atoms (C2—C7, C9—C14 and C2B—C7B, C9B—C14B), oxime oxygen atom (O4 and O4b), and oxime nitrogen atoms (N1, N2 and N1B, N2B) of the salicylhydroximate ligands, equivalent atoms were constrained to have pairwise identical anisotropic displacement parameters (ADPs). The ADPs of the sodium ions (Na1 and Na1B) were also constrained to be identical. For the coordinating DMF molecules, the nitrogen atoms (N3 and N3B, N4 and N4B, and N5 and N5B) have nearly the same atom positions leading to highly correlated thermal parameters. To avoid correlation of the thermal parameters, the ADPs of equivalent nitrogen atoms in the DMF molecules were constrained to be identical. In addition, carbon, oxygen, and chlorine atoms of the chloroacetate and carbon, oxygen, and nitrogen atoms of the coordinating DMF molecules were restrained to have similar *U_ij_* components of the ADPs (e.s.d. = 0.04 Angstrom squared; SIMU restraint in Shexl). Anisotropic displacement parameters of all atoms in the minor moiety of the coordinating DMF molecule associated with N5B were restrained using an enhanced rigid bond restraint for the 1,2- and 1,3 distances [RIGU command in *SHELXL*, e.s.d. = 0.004 Angstrom squared for both 1,2- and 1,3 distances [Thorn, Dittrich & Sheldrick, Acta Cryst. A68 (2012) 448–451]. Additionally, the following sodium-oxygen bond distances were restrained to be similar (e.s.d. 0.02 Angstrom): Na1—O1 and Na1B—O1B, Na1—O4 and Na1B—O4B, Na1—O8 and Na1B—O8B, and Na1—O11 and Na1B—O11B. Subject to these conditions, the occupancy ratio of the disordered metallacrown and associated anion and solvent molecules refined to 0.8783 (7) to 0.1217 (7).A lattice DMF molecule, associated with N6, is disordered over two sites with different orientations. The geometries of the two DMF molecules were restrained to be similar to each other (SAME command in *SHELXL*, e.s.d. = 0.02 Angstrom). The nitrogen atoms (N6 and N6B) have nearly the same atom positions leading to highly correlated thermal parameters. To avoid correlation of the thermal parameters, the ADPs of equivalent atoms were constrained to be identical. In addition, carbon, oxygen, and nitrogen atoms of the DMF molecule were restrained to have similar *U_ij_* components of the ADPs (e.s.d. = 0.04 Angstrom squared; SIMU restraint in Shexltl). Subject to these restraints, the occupancy ratio of the disordered DMF molecule refined to 0.615 (5) to 0.385 (5).All hydrogen atoms were placed in calculated positions and refined as riding on their carrier atoms with C—H distances of 0.95 Angstrom for *sp*^2^ carbon atoms and 0.98 Angstrom for methyl carbon atoms. The *U*_iso_ values for hydrogen atoms were set to a multiple of the value of the carrying carbon atom (1.2 times for *sp*^2^-hybridized carbon atoms or 1.5 times for methyl carbon atoms and water oxygen atoms). Major moiety methyl H atoms were allowed to rotate, but not to tip (AFIX 137 command in *SHELXL*). For the minor moiety methyl H atoms the C—N—C—H dihedral angle were constrained as implemented in the AFIX 33 command in *SHELXL*.
Geometry. All e.s.d.'s (except the e.s.d. in the dihedral angle between two l.s. planes) are estimated using the full covariance matrix. The cell e.s.d.'s are taken into account individually in the estimation of e.s.d.'s in distances, angles and torsion angles; correlations between e.s.d.'s in cell parameters are only used when they are defined by crystal symmetry. An approximate (isotropic) treatment of cell e.s.d.'s is used for estimating e.s.d.'s involving l.s. planes.

### Fractional atomic coordinates and isotropic or equivalent isotropic displacement parameters (Å^2^)

**Table:**

	*x*	*y*	*z*	*U*_iso_*/*U*_eq_	Occ. (<1)
Mn1	0.38268 (2)	0.19054 (2)	0.48930 (2)	0.01265 (6)	0.8783 (7)
O1	0.48341 (8)	0.12275 (8)	0.46364 (8)	0.0142 (2)	0.8783 (7)
N1	0.56296 (11)	0.17545 (11)	0.45959 (11)	0.0136 (3)	0.8783 (7)
O2	0.46659 (9)	0.29180 (9)	0.47696 (8)	0.0149 (2)	0.8783 (7)
C1	0.54901 (12)	0.26267 (12)	0.46741 (10)	0.0139 (3)	0.8783 (7)
C2	0.62548 (13)	0.32778 (13)	0.46514 (10)	0.0136 (3)	0.8783 (7)
C3	0.60694 (14)	0.41995 (14)	0.47522 (11)	0.0175 (3)	0.8783 (7)
H3	0.5461	0.4376	0.4829	0.021*	0.8783 (7)
C4	0.67519 (16)	0.48657 (13)	0.47437 (13)	0.0205 (4)	0.8783 (7)
H4	0.6622	0.5488	0.4826	0.025*	0.8783 (7)
C5	0.76359 (16)	0.45917 (14)	0.46097 (13)	0.0191 (4)	0.8783 (7)
H5	0.8110	0.5035	0.4595	0.023*	0.8783 (7)
C6	0.78298 (14)	0.36865 (14)	0.44988 (12)	0.0173 (3)	0.8783 (7)
H6	0.8430	0.3520	0.4395	0.021*	0.8783 (7)
C7	0.71570 (14)	0.30050 (12)	0.45363 (11)	0.0160 (3)	0.8783 (7)
O3	0.74222 (10)	0.21471 (9)	0.44710 (10)	0.0231 (3)	0.8783 (7)
Mn2	0.67670 (2)	0.10591 (2)	0.44892 (2)	0.01554 (6)	0.8783 (7)
O4	0.61678 (8)	−0.00007 (8)	0.47493 (8)	0.0141 (2)	0.8783 (7)
N2	0.67577 (13)	−0.07555 (11)	0.48657 (12)	0.0138 (3)	0.8783 (7)
O5	0.78646 (9)	0.02562 (9)	0.46738 (9)	0.0189 (3)	0.8783 (7)
C8	0.76264 (12)	−0.05796 (12)	0.48044 (11)	0.0149 (3)	0.8783 (7)
C9	0.83091 (13)	−0.13234 (13)	0.48468 (11)	0.0153 (3)	0.8783 (7)
C10	0.92497 (15)	−0.10935 (14)	0.48404 (13)	0.0209 (4)	0.8783 (7)
H10	0.9423	−0.0470	0.4849	0.025*	0.8783 (7)
C11	0.99360 (13)	−0.17516 (16)	0.48220 (13)	0.0209 (4)	0.8783 (7)
H11	1.0571	−0.1584	0.4820	0.025*	0.8783 (7)
C12	0.96701 (15)	−0.26643 (15)	0.48067 (13)	0.0203 (4)	0.8783 (7)
H12	1.0127	−0.3124	0.4788	0.024*	0.8783 (7)
C13	0.87446 (15)	−0.29064 (14)	0.48182 (12)	0.0188 (4)	0.8783 (7)
H13	0.8580	−0.3532	0.4814	0.023*	0.8783 (7)
C14	0.80446 (12)	−0.22480 (14)	0.48360 (11)	0.0145 (3)	0.8783 (7)
O6	0.71625 (9)	−0.25443 (9)	0.48067 (8)	0.0174 (2)	0.8783 (7)
O7	0.64832 (11)	0.08643 (10)	0.31936 (9)	0.0242 (3)	0.8783 (7)
C15	0.57616 (16)	0.05246 (14)	0.27175 (12)	0.0207 (4)	0.8783 (7)
O8	0.50242 (16)	0.02543 (15)	0.28940 (14)	0.0240 (4)	0.8783 (7)
C16	0.5892 (2)	0.05038 (19)	0.18215 (16)	0.0289 (5)	0.8783 (7)
H16A	0.6531	0.0265	0.1814	0.035*	0.8783 (7)
H16B	0.5861	0.1135	0.1609	0.035*	0.8783 (7)
Cl1	0.50475 (7)	−0.01609 (8)	0.11496 (5)	0.03164 (18)	0.8783 (7)
O9	0.30672 (10)	0.19411 (10)	0.36021 (8)	0.0209 (3)	0.8783 (7)
C17	0.3478 (3)	0.1934 (4)	0.30087 (16)	0.0224 (6)	0.8783 (7)
H17	0.4150	0.1925	0.3132	0.027*	0.8783 (7)
N3	0.30499 (17)	0.1939 (2)	0.22187 (14)	0.0249 (5)	0.8783 (7)
C18	0.2023 (3)	0.1873 (3)	0.1979 (2)	0.0359 (8)	0.8783 (7)
H18A	0.1765	0.2452	0.1741	0.054*	0.8783 (7)
H18B	0.1761	0.1734	0.2466	0.054*	0.8783 (7)
H18C	0.1853	0.1388	0.1570	0.054*	0.8783 (7)
C19	0.3584 (3)	0.1960 (2)	0.15659 (18)	0.0419 (7)	0.8783 (7)
H19A	0.3514	0.1377	0.1273	0.063*	0.8783 (7)
H19B	0.4254	0.2067	0.1805	0.063*	0.8783 (7)
H19C	0.3344	0.2451	0.1179	0.063*	0.8783 (7)
O10	0.52243 (13)	−0.17570 (11)	0.37299 (12)	0.0215 (3)	0.8783 (7)
C20	0.51607 (16)	−0.19688 (14)	0.30007 (12)	0.0254 (4)	0.8783 (7)
H20	0.4969	−0.1510	0.2597	0.031*	0.8783 (7)
N4	0.5339 (5)	−0.2784 (3)	0.27374 (16)	0.0343 (6)	0.8783 (7)
C21	0.56354 (19)	−0.35315 (15)	0.33013 (14)	0.0335 (5)	0.8783 (7)
H21A	0.5350	−0.4098	0.3055	0.050*	0.8783 (7)
H21B	0.5431	−0.3417	0.3821	0.050*	0.8783 (7)
H21C	0.6326	−0.3585	0.3409	0.050*	0.8783 (7)
C22	0.5273 (3)	−0.2964 (2)	0.18536 (15)	0.0533 (8)	0.8783 (7)
H22A	0.5082	−0.2407	0.1538	0.080*	0.8783 (7)
H22B	0.4803	−0.3441	0.1672	0.080*	0.8783 (7)
H22C	0.5890	−0.3163	0.1762	0.080*	0.8783 (7)
O11	0.30119 (13)	−0.08465 (15)	0.36626 (13)	0.0387 (4)	0.8783 (7)
C23	0.22369 (18)	−0.0752 (2)	0.31880 (17)	0.0360 (6)	0.8783 (7)
H23	0.1693	−0.0724	0.3426	0.043*	0.8783 (7)
N5	0.21038 (14)	−0.06854 (16)	0.23725 (14)	0.0315 (5)	0.8783 (7)
C24	0.2894 (2)	−0.0710 (3)	0.1945 (2)	0.0418 (7)	0.8783 (7)
H24A	0.2945	−0.1321	0.1721	0.063*	0.8783 (7)
H24B	0.3482	−0.0560	0.2334	0.063*	0.8783 (7)
H24C	0.2785	−0.0267	0.1494	0.063*	0.8783 (7)
C25	0.1168 (2)	−0.0650 (4)	0.1845 (3)	0.0375 (8)	0.8783 (7)
H25A	0.1091	−0.0074	0.1541	0.056*	0.8783 (7)
H25B	0.0688	−0.0694	0.2185	0.056*	0.8783 (7)
H25C	0.1093	−0.1158	0.1454	0.056*	0.8783 (7)
O12	0.5406 (3)	0.3039 (3)	0.2524 (2)	0.0614 (12)	0.615 (5)
C26	0.6199 (6)	0.2984 (7)	0.2465 (6)	0.054 (3)	0.615 (5)
H26	0.6445	0.2384	0.2526	0.065*	0.615 (5)
N6	0.6808 (5)	0.3594 (9)	0.2330 (7)	0.0392 (13)	0.615 (5)
C27	0.6502 (4)	0.4538 (4)	0.2260 (4)	0.0418 (12)	0.615 (5)
H27A	0.6884	0.4876	0.1936	0.063*	0.615 (5)
H27B	0.6583	0.4806	0.2811	0.063*	0.615 (5)
H27C	0.5834	0.4567	0.1984	0.063*	0.615 (5)
C28	0.7754 (4)	0.3437 (4)	0.2271 (4)	0.0364 (10)	0.615 (5)
H28A	0.7837	0.2794	0.2150	0.055*	0.615 (5)
H28B	0.8175	0.3597	0.2795	0.055*	0.615 (5)
H28C	0.7908	0.3812	0.1828	0.055*	0.615 (5)
Na1	0.45845 (14)	−0.03121 (17)	0.40525 (15)	0.0144 (3)	0.8783 (7)
Mn1B	0.53392 (15)	−0.21852 (16)	0.52400 (15)	0.0230 (6)	0.1217 (7)
O1B	0.5784 (6)	−0.1004 (6)	0.5135 (8)	0.031 (3)	0.1217 (7)
N1B	0.6691 (9)	−0.0984 (7)	0.4957 (11)	0.0136 (3)	0.1217 (7)
O2B	0.6646 (6)	−0.2498 (6)	0.5139 (7)	0.024 (2)	0.1217 (7)
C1B	0.7104 (8)	−0.1782 (8)	0.4991 (8)	0.022 (3)	0.1217 (7)
C2B	0.8093 (8)	−0.1823 (9)	0.4893 (8)	0.0136 (3)	0.1217 (7)
C3B	0.8439 (10)	−0.2697 (9)	0.4897 (9)	0.0175 (3)	0.1217 (7)
H3B	0.8046	−0.3195	0.4969	0.021*	0.1217 (7)
C4B	0.9345 (11)	−0.2855 (10)	0.4799 (10)	0.0205 (4)	0.1217 (7)
H4B	0.9562	−0.3458	0.4751	0.025*	0.1217 (7)
C5B	0.9925 (11)	−0.2138 (10)	0.4770 (9)	0.0191 (4)	0.1217 (7)
H5B	1.0567	−0.2240	0.4742	0.023*	0.1217 (7)
C6B	0.9591 (10)	−0.1264 (9)	0.4780 (9)	0.0173 (3)	0.1217 (7)
H6B	1.0005	−0.0769	0.4749	0.021*	0.1217 (7)
C7B	0.8662 (9)	−0.1092 (8)	0.4833 (9)	0.0160 (3)	0.1217 (7)
O3B	0.8362 (7)	−0.0222 (7)	0.4853 (9)	0.036 (3)	0.1217 (7)
Mn2B	0.71495 (16)	0.02477 (16)	0.47156 (17)	0.0271 (6)	0.1217 (7)
O4B	0.5938 (5)	0.0704 (5)	0.4741 (5)	0.0141 (2)	0.1217 (7)
N2B	0.5901 (8)	0.1646 (8)	0.4702 (10)	0.0138 (3)	0.1217 (7)
O5B	0.7458 (6)	0.1546 (6)	0.4723 (6)	0.020 (2)	0.1217 (7)
C8B	0.6726 (8)	0.2053 (6)	0.4709 (8)	0.016 (2)	0.1217 (7)
C9B	0.6754 (9)	0.3048 (7)	0.4653 (9)	0.0153 (3)	0.1217 (7)
C10B	0.7640 (10)	0.3387 (10)	0.4594 (10)	0.0209 (4)	0.1217 (7)
H10B	0.8143	0.2980	0.4567	0.025*	0.1217 (7)
C11B	0.7784 (11)	0.4302 (11)	0.4576 (12)	0.0209 (4)	0.1217 (7)
H11B	0.8386	0.4530	0.4530	0.025*	0.1217 (7)
C12B	0.7064 (10)	0.4895 (11)	0.4625 (11)	0.0203 (4)	0.1217 (7)
H12B	0.7170	0.5532	0.4626	0.024*	0.1217 (7)
C13B	0.6189 (10)	0.4560 (9)	0.4672 (10)	0.0188 (4)	0.1217 (7)
H13B	0.5687	0.4972	0.4694	0.023*	0.1217 (7)
C14B	0.6026 (8)	0.3642 (9)	0.4689 (8)	0.0145 (3)	0.1217 (7)
O6B	0.5143 (6)	0.3336 (7)	0.4750 (7)	0.024 (2)	0.1217 (7)
O7B	0.6868 (9)	0.0206 (12)	0.3389 (8)	0.050 (4)	0.1217 (7)
C15B	0.6092 (12)	0.012 (2)	0.2877 (10)	0.053 (5)	0.1217 (7)
O8B	0.5302 (12)	−0.0067 (17)	0.2989 (12)	0.056 (6)	0.1217 (7)
C16B	0.6179 (15)	0.025 (2)	0.1987 (9)	0.047 (6)	0.1217 (7)
H16C	0.6697	−0.0144	0.1888	0.056*	0.1217 (7)
H16D	0.6387	0.0890	0.1933	0.056*	0.1217 (7)
Cl1B	0.5184 (11)	0.0057 (9)	0.1177 (8)	0.089 (5)	0.1217 (7)
O9B	0.4183 (9)	0.2152 (10)	0.3374 (8)	0.041 (3)	0.1217 (7)
C17B	0.3316 (17)	0.197 (4)	0.3084 (17)	0.051 (9)	0.1217 (7)
H17B	0.2927	0.1942	0.3480	0.061*	0.1217 (7)
N3B	0.2873 (14)	0.182 (2)	0.2305 (12)	0.0249 (5)	0.1217 (7)
C18B	0.331 (2)	0.162 (3)	0.1623 (17)	0.082 (11)	0.1217 (7)
H18D	0.3423	0.2179	0.1345	0.123*	0.1217 (7)
H18E	0.2887	0.1221	0.1234	0.123*	0.1217 (7)
H18F	0.3910	0.1303	0.1825	0.123*	0.1217 (7)
C19B	0.1873 (19)	0.208 (3)	0.207 (3)	0.076 (14)	0.1217 (7)
H19D	0.1773	0.2458	0.1574	0.115*	0.1217 (7)
H19E	0.1695	0.2428	0.2523	0.115*	0.1217 (7)
H19F	0.1483	0.1533	0.1966	0.115*	0.1217 (7)
O10B	0.5012 (15)	−0.2125 (15)	0.3797 (10)	0.061 (5)	0.1217 (7)
C20B	0.5396 (19)	−0.2697 (16)	0.3421 (11)	0.063 (6)	0.1217 (7)
H20B	0.5840	−0.3088	0.3755	0.076*	0.1217 (7)
N4B	0.527 (4)	−0.283 (3)	0.2608 (13)	0.0343 (6)	0.1217 (7)
C21B	0.483 (3)	−0.2153 (19)	0.2022 (16)	0.090 (10)	0.1217 (7)
H21D	0.5216	−0.2070	0.1603	0.135*	0.1217 (7)
H21E	0.4795	−0.1576	0.2310	0.135*	0.1217 (7)
H21F	0.4196	−0.2350	0.1757	0.135*	0.1217 (7)
C22B	0.552 (3)	−0.371 (2)	0.228 (2)	0.119 (12)	0.1217 (7)
H22D	0.4952	−0.3996	0.1949	0.179*	0.1217 (7)
H22E	0.5791	−0.4116	0.2739	0.179*	0.1217 (7)
H22F	0.5985	−0.3612	0.1933	0.179*	0.1217 (7)
O11B	0.2936 (11)	−0.0372 (12)	0.3615 (12)	0.064 (5)	0.1217 (7)
C23B	0.2075 (15)	−0.039 (2)	0.3243 (12)	0.060 (5)	0.1217 (7)
H23B	0.1600	−0.0255	0.3547	0.072*	0.1217 (7)
N5B	0.1823 (11)	−0.0571 (14)	0.2475 (10)	0.0315 (5)	0.1217 (7)
C24B	0.2643 (17)	−0.074 (3)	0.2090 (19)	0.061 (8)	0.1217 (7)
H24D	0.2432	−0.1046	0.1560	0.091*	0.1217 (7)
H24E	0.3105	−0.1119	0.2455	0.091*	0.1217 (7)
H24F	0.2937	−0.0156	0.1999	0.091*	0.1217 (7)
C25B	0.0969 (18)	−0.054 (4)	0.188 (2)	0.057 (10)	0.1217 (7)
H25D	0.0704	0.0070	0.1854	0.086*	0.1217 (7)
H25E	0.0517	−0.0976	0.2033	0.086*	0.1217 (7)
H25F	0.1094	−0.0710	0.1339	0.086*	0.1217 (7)
O12B	0.5372 (4)	0.4724 (3)	0.1987 (3)	0.0521 (16)	0.385 (5)
C26B	0.6174 (7)	0.4520 (8)	0.1999 (6)	0.055 (3)	0.385 (5)
H26B	0.6549	0.4972	0.1811	0.066*	0.385 (5)
N6B	0.6610 (10)	0.3739 (15)	0.2242 (13)	0.0392 (13)	0.385 (5)
C27B	0.6083 (12)	0.2978 (7)	0.2465 (9)	0.059 (6)	0.385 (5)
H27D	0.5696	0.3182	0.2852	0.088*	0.385 (5)
H27E	0.6523	0.2506	0.2726	0.088*	0.385 (5)
H27F	0.5672	0.2731	0.1969	0.088*	0.385 (5)
C28B	0.7558 (11)	0.3431 (16)	0.2255 (12)	0.164 (10)	0.385 (5)
H28D	0.7663	0.3401	0.1690	0.246*	0.385 (5)
H28E	0.7647	0.2825	0.2506	0.246*	0.385 (5)
H28F	0.8010	0.3856	0.2578	0.246*	0.385 (5)
Na1B	0.4601 (11)	−0.0206 (15)	0.4096 (11)	0.0144 (3)	0.1217 (7)

### Atomic displacement parameters (Å^2^)

**Table:**

	*U*^11^	*U*^22^	*U*^33^	*U*^12^	*U*^13^	*U*^23^
Mn1	0.01043 (12)	0.01275 (12)	0.01532 (11)	−0.00049 (9)	0.00393 (9)	−0.00080 (9)
O1	0.0105 (5)	0.0142 (6)	0.0192 (6)	−0.0027 (4)	0.0058 (4)	−0.0008 (4)
N1	0.0092 (7)	0.0135 (7)	0.0184 (7)	−0.0026 (6)	0.0037 (6)	0.0004 (5)
O2	0.0128 (6)	0.0144 (6)	0.0181 (6)	−0.0003 (5)	0.0049 (5)	0.0004 (4)
C1	0.0134 (8)	0.0147 (7)	0.0130 (7)	−0.0007 (6)	0.0015 (6)	0.0001 (6)
C2	0.0141 (8)	0.0123 (7)	0.0143 (7)	−0.0015 (6)	0.0022 (6)	−0.0002 (6)
C3	0.0193 (9)	0.0133 (8)	0.0198 (8)	−0.0013 (7)	0.0035 (7)	−0.0016 (7)
C4	0.0241 (11)	0.0139 (8)	0.0233 (9)	−0.0023 (8)	0.0047 (8)	−0.0006 (7)
C5	0.0231 (11)	0.0142 (9)	0.0203 (8)	−0.0071 (8)	0.0048 (7)	0.0000 (7)
C6	0.0151 (8)	0.0167 (9)	0.0199 (8)	−0.0038 (7)	0.0032 (7)	0.0009 (7)
C7	0.0146 (8)	0.0141 (8)	0.0185 (8)	−0.0016 (7)	0.0017 (7)	0.0005 (6)
O3	0.0154 (6)	0.0137 (6)	0.0424 (8)	−0.0033 (5)	0.0106 (6)	−0.0013 (6)
Mn2	0.01122 (12)	0.01245 (12)	0.02429 (13)	−0.00198 (9)	0.00678 (10)	−0.00102 (10)
O4	0.0106 (5)	0.0126 (5)	0.0194 (6)	0.0003 (4)	0.0038 (4)	0.0012 (4)
N2	0.0117 (7)	0.0118 (7)	0.0183 (8)	0.0000 (6)	0.0038 (5)	0.0001 (6)
O5	0.0115 (6)	0.0164 (6)	0.0294 (7)	−0.0023 (5)	0.0058 (5)	−0.0011 (5)
C8	0.0115 (7)	0.0164 (8)	0.0164 (7)	−0.0025 (6)	0.0021 (6)	−0.0022 (6)
C9	0.0100 (8)	0.0165 (8)	0.0195 (8)	−0.0018 (6)	0.0028 (6)	−0.0030 (6)
C10	0.0124 (9)	0.0204 (9)	0.0294 (10)	−0.0012 (7)	0.0033 (7)	−0.0027 (7)
C11	0.0107 (8)	0.0226 (9)	0.0297 (10)	−0.0021 (8)	0.0049 (7)	−0.0019 (8)
C12	0.0137 (9)	0.0224 (10)	0.0250 (9)	0.0023 (7)	0.0042 (8)	−0.0052 (7)
C13	0.0147 (9)	0.0185 (9)	0.0230 (9)	0.0016 (7)	0.0037 (7)	−0.0036 (7)
C14	0.0125 (8)	0.0157 (8)	0.0157 (7)	0.0002 (7)	0.0036 (6)	−0.0024 (6)
O6	0.0128 (6)	0.0160 (6)	0.0247 (6)	−0.0020 (5)	0.0066 (5)	−0.0047 (5)
O7	0.0267 (7)	0.0260 (7)	0.0218 (7)	−0.0027 (6)	0.0093 (6)	0.0023 (6)
C15	0.0280 (11)	0.0176 (9)	0.0180 (8)	0.0052 (8)	0.0085 (8)	0.0034 (7)
O8	0.0250 (10)	0.0292 (9)	0.0193 (8)	−0.0006 (7)	0.0082 (8)	0.0026 (6)
C16	0.0369 (15)	0.0344 (14)	0.0173 (11)	−0.0031 (11)	0.0102 (10)	0.0017 (9)
Cl1	0.0360 (3)	0.0417 (5)	0.0176 (2)	0.0055 (3)	0.0061 (2)	−0.0036 (2)
O9	0.0189 (7)	0.0273 (7)	0.0159 (6)	0.0006 (5)	0.0022 (5)	0.0007 (5)
C17	0.0214 (13)	0.0291 (13)	0.0151 (9)	0.0059 (12)	0.0002 (9)	−0.0028 (9)
N3	0.0268 (12)	0.0304 (12)	0.0163 (9)	0.0042 (9)	0.0020 (8)	−0.0004 (8)
C18	0.0307 (16)	0.042 (2)	0.0287 (12)	−0.0022 (15)	−0.0091 (11)	0.0058 (11)
C19	0.050 (2)	0.0576 (19)	0.0213 (11)	0.0117 (15)	0.0139 (12)	0.0021 (12)
O10	0.0248 (8)	0.0208 (7)	0.0176 (7)	−0.0027 (6)	0.0015 (6)	−0.0011 (6)
C20	0.0302 (11)	0.0241 (10)	0.0198 (8)	0.0007 (8)	0.0000 (8)	−0.0004 (7)
N4	0.0543 (18)	0.0287 (10)	0.0173 (12)	0.0036 (11)	0.0016 (16)	−0.0043 (10)
C21	0.0516 (15)	0.0197 (10)	0.0267 (10)	0.0012 (9)	0.0022 (10)	−0.0030 (8)
C22	0.094 (3)	0.0424 (15)	0.0203 (10)	0.0123 (16)	0.0050 (13)	−0.0054 (10)
O11	0.0237 (8)	0.0439 (11)	0.0427 (10)	−0.0017 (8)	−0.0069 (7)	−0.0132 (9)
C23	0.0228 (11)	0.0398 (15)	0.0442 (14)	−0.0028 (10)	0.0039 (10)	−0.0168 (11)
N5	0.0170 (10)	0.0357 (11)	0.0400 (11)	0.0012 (9)	0.0020 (8)	−0.0107 (8)
C24	0.0299 (16)	0.0556 (19)	0.0413 (16)	0.0018 (14)	0.0102 (12)	−0.0035 (13)
C25	0.0255 (16)	0.0374 (16)	0.0443 (16)	0.0018 (15)	−0.0048 (13)	−0.0028 (12)
O12	0.048 (2)	0.092 (3)	0.0470 (19)	−0.0248 (19)	0.0183 (16)	0.0003 (17)
C26	0.039 (3)	0.091 (6)	0.033 (4)	−0.012 (3)	0.008 (3)	−0.011 (3)
N6	0.038 (4)	0.052 (4)	0.027 (3)	−0.001 (3)	0.006 (3)	−0.004 (2)
C27	0.044 (3)	0.036 (2)	0.048 (3)	−0.001 (2)	0.014 (2)	−0.010 (2)
C28	0.0304 (19)	0.051 (2)	0.031 (2)	−0.0138 (18)	0.0140 (16)	−0.0121 (18)
Na1	0.0126 (3)	0.0148 (8)	0.0162 (4)	−0.0019 (3)	0.0036 (3)	−0.0017 (4)
Mn1B	0.0105 (9)	0.0238 (11)	0.0355 (12)	−0.0024 (8)	0.0064 (8)	0.0051 (9)
O1B	0.013 (5)	0.021 (5)	0.065 (8)	0.008 (4)	0.020 (5)	0.009 (5)
N1B	0.0092 (7)	0.0135 (7)	0.0184 (7)	−0.0026 (6)	0.0037 (6)	0.0004 (5)
O2B	0.022 (5)	0.013 (4)	0.038 (6)	0.001 (4)	0.007 (4)	0.001 (4)
C1B	0.014 (6)	0.035 (8)	0.018 (6)	0.000 (5)	0.004 (5)	0.004 (5)
C2B	0.0141 (8)	0.0123 (7)	0.0143 (7)	−0.0015 (6)	0.0022 (6)	−0.0002 (6)
C3B	0.0193 (9)	0.0133 (8)	0.0198 (8)	−0.0013 (7)	0.0035 (7)	−0.0016 (7)
C4B	0.0241 (11)	0.0139 (8)	0.0233 (9)	−0.0023 (8)	0.0047 (8)	−0.0006 (7)
C5B	0.0231 (11)	0.0142 (9)	0.0203 (8)	−0.0071 (8)	0.0048 (7)	0.0000 (7)
C6B	0.0151 (8)	0.0167 (9)	0.0199 (8)	−0.0038 (7)	0.0032 (7)	0.0009 (7)
C7B	0.0146 (8)	0.0141 (8)	0.0185 (8)	−0.0016 (7)	0.0017 (7)	0.0005 (6)
O3B	0.022 (6)	0.021 (5)	0.071 (9)	−0.005 (5)	0.024 (6)	−0.004 (5)
Mn2B	0.0174 (11)	0.0202 (11)	0.0469 (15)	−0.0021 (8)	0.0138 (10)	0.0014 (10)
O4B	0.0106 (5)	0.0126 (5)	0.0194 (6)	0.0003 (4)	0.0038 (4)	0.0012 (4)
N2B	0.0117 (7)	0.0118 (7)	0.0183 (8)	0.0000 (6)	0.0038 (5)	0.0001 (6)
O5B	0.012 (4)	0.027 (5)	0.022 (5)	−0.008 (4)	0.003 (4)	−0.007 (4)
C8B	0.018 (6)	0.010 (5)	0.021 (6)	−0.003 (4)	0.004 (5)	0.003 (4)
C9B	0.0100 (8)	0.0165 (8)	0.0195 (8)	−0.0018 (6)	0.0028 (6)	−0.0030 (6)
C10B	0.0124 (9)	0.0204 (9)	0.0294 (10)	−0.0012 (7)	0.0033 (7)	−0.0027 (7)
C11B	0.0107 (8)	0.0226 (9)	0.0297 (10)	−0.0021 (8)	0.0049 (7)	−0.0019 (8)
C12B	0.0137 (9)	0.0224 (10)	0.0250 (9)	0.0023 (7)	0.0042 (8)	−0.0052 (7)
C13B	0.0147 (9)	0.0185 (9)	0.0230 (9)	0.0016 (7)	0.0037 (7)	−0.0036 (7)
C14B	0.0125 (8)	0.0157 (8)	0.0157 (7)	0.0002 (7)	0.0036 (6)	−0.0024 (6)
O6B	0.008 (4)	0.020 (5)	0.045 (6)	0.005 (4)	0.011 (4)	−0.001 (4)
O7B	0.043 (7)	0.076 (10)	0.032 (6)	−0.019 (7)	0.011 (6)	−0.013 (7)
C15B	0.049 (11)	0.078 (13)	0.035 (9)	−0.008 (10)	0.015 (8)	0.005 (10)
O8B	0.042 (12)	0.097 (19)	0.029 (8)	−0.036 (11)	0.005 (8)	−0.003 (11)
C16B	0.045 (12)	0.062 (14)	0.025 (9)	−0.011 (10)	−0.012 (8)	0.018 (9)
Cl1B	0.120 (10)	0.048 (6)	0.115 (9)	−0.002 (5)	0.064 (7)	−0.001 (5)
O9B	0.030 (6)	0.049 (7)	0.041 (7)	0.004 (5)	0.003 (5)	−0.014 (6)
C17B	0.021 (11)	0.046 (13)	0.079 (16)	0.004 (10)	−0.006 (11)	−0.003 (14)
N3B	0.0268 (12)	0.0304 (12)	0.0163 (9)	0.0042 (9)	0.0020 (8)	−0.0004 (8)
C18B	0.049 (17)	0.14 (3)	0.061 (17)	0.038 (17)	0.025 (13)	0.056 (18)
C19B	0.035 (14)	0.06 (2)	0.12 (3)	0.023 (13)	−0.026 (16)	−0.003 (18)
O10B	0.070 (12)	0.095 (14)	0.016 (6)	−0.009 (11)	0.000 (7)	0.001 (9)
C20B	0.078 (13)	0.063 (12)	0.038 (9)	0.009 (11)	−0.014 (10)	0.000 (9)
N4B	0.0543 (18)	0.0287 (10)	0.0173 (12)	0.0036 (11)	0.0016 (16)	−0.0043 (10)
C21B	0.13 (2)	0.072 (18)	0.056 (15)	−0.014 (18)	−0.013 (16)	0.007 (13)
C22B	0.20 (3)	0.09 (2)	0.070 (19)	0.00 (2)	0.02 (2)	−0.021 (17)
O11B	0.061 (8)	0.035 (8)	0.076 (9)	−0.007 (7)	−0.033 (6)	0.016 (8)
C23B	0.058 (8)	0.064 (13)	0.049 (5)	0.005 (8)	−0.009 (5)	−0.020 (6)
N5B	0.0170 (10)	0.0357 (11)	0.0400 (11)	0.0012 (9)	0.0020 (8)	−0.0107 (8)
C24B	0.033 (8)	0.078 (19)	0.067 (11)	0.005 (8)	0.005 (7)	−0.019 (11)
C25B	0.019 (7)	0.09 (3)	0.055 (10)	0.012 (8)	−0.012 (7)	−0.024 (11)
O12B	0.062 (3)	0.047 (3)	0.053 (3)	0.008 (2)	0.026 (2)	−0.012 (2)
C26B	0.056 (6)	0.063 (5)	0.046 (5)	−0.003 (5)	0.008 (4)	−0.024 (4)
N6B	0.038 (4)	0.052 (4)	0.027 (3)	−0.001 (3)	0.006 (3)	−0.004 (2)
C27B	0.126 (15)	0.018 (4)	0.028 (6)	−0.020 (6)	0.007 (7)	0.005 (4)
C28B	0.114 (14)	0.31 (2)	0.063 (9)	0.087 (14)	0.002 (9)	−0.069 (12)
Na1B	0.0126 (3)	0.0148 (8)	0.0162 (4)	−0.0019 (3)	0.0036 (3)	−0.0017 (4)

### Geometric parameters (Å, º)

**Table:**

Mn1—O6^i^	1.8616 (13)	Mn1B—O1B	1.873 (9)
Mn1—O1	1.8831 (12)	Mn1B—N2B^i^	1.980 (12)
Mn1—O2	1.9574 (13)	Mn1B—O2B	1.983 (9)
Mn1—N2^i^	1.9678 (15)	Mn1B—O9B^i^	2.258 (13)
Mn1—O9	2.1945 (14)	Mn1B—O10B	2.342 (16)
Mn1—O10^i^	2.4179 (19)	Mn1B—Na1B	3.52 (2)
Mn1—Na1^i^	3.488 (3)	Mn1B—Na1B^i^	3.68 (2)
O1—N1	1.3991 (19)	O1B—N1B	1.401 (13)
O1—Na1	2.460 (3)	O1B—Na1B^i^	2.32 (2)
O1—Na1^i^	2.547 (3)	O1B—Na1B	2.460 (18)
N1—C1	1.309 (2)	N1B—C1B	1.312 (14)
N1—Mn2	1.9740 (16)	N1B—Mn2B	1.997 (10)
O2—C1	1.305 (2)	O2B—C1B	1.295 (12)
C1—C2	1.468 (2)	C1B—C2B	1.471 (13)
C2—C3	1.399 (3)	C2B—C7B	1.369 (14)
C2—C7	1.414 (3)	C2B—C3B	1.379 (14)
C3—C4	1.392 (3)	C3B—C4B	1.371 (15)
C3—H3	0.9500	C3B—H3B	0.9500
C4—C5	1.400 (3)	C4B—C5B	1.354 (14)
C4—H4	0.9500	C4B—H4B	0.9500
C5—C6	1.381 (3)	C5B—C6B	1.374 (15)
C5—H5	0.9500	C5B—H5B	0.9500
C6—C7	1.406 (3)	C6B—C7B	1.388 (14)
C6—H6	0.9500	C6B—H6B	0.9500
C7—O3	1.330 (2)	C7B—O3B	1.353 (12)
O3—Mn2	1.8627 (14)	O3B—Mn2B	1.853 (9)
Mn2—O4	1.8756 (12)	Mn2B—O4B	1.882 (8)
Mn2—O5	1.9501 (14)	Mn2B—O5B	1.960 (9)
Mn2—O7	2.1202 (15)	Mn2B—O7B	2.152 (13)
Mn2—Na1^i^	3.577 (3)	Mn2B—Na1B^i^	3.51 (2)
Mn2—Na1	3.6869 (19)	Mn2B—Na1B	3.674 (14)
O4—N2	1.389 (2)	O4B—N2B	1.387 (12)
O4—Na1	2.385 (2)	O4B—Na1B^i^	2.34 (2)
O4—Na1^i^	2.494 (3)	O4B—Na1B	2.412 (15)
N2—C8	1.306 (2)	N2B—C8B	1.331 (13)
N2—Mn1^i^	1.9679 (15)	N2B—Mn1B^i^	1.980 (12)
O5—C8	1.307 (2)	O5B—C8B	1.291 (12)
C8—C9	1.465 (2)	C8B—C9B	1.468 (12)
C9—C10	1.402 (3)	C9B—C14B	1.378 (14)
C9—C14	1.412 (3)	C9B—C10B	1.395 (14)
C10—C11	1.390 (3)	C10B—C11B	1.363 (15)
C10—H10	0.9500	C10B—H10B	0.9500
C11—C12	1.395 (3)	C11B—C12B	1.373 (15)
C11—H11	0.9500	C11B—H11B	0.9500
C12—C13	1.387 (3)	C12B—C13B	1.373 (14)
C12—H12	0.9500	C12B—H12B	0.9500
C13—C14	1.405 (3)	C13B—C14B	1.373 (14)
C13—H13	0.9500	C13B—H13B	0.9500
C14—O6	1.338 (2)	C14B—O6B	1.375 (12)
O6—Mn1^i^	1.8616 (13)	O6B—Mn1B^i^	1.832 (10)
O7—C15	1.273 (3)	O7B—C15B	1.266 (15)
C15—O8	1.227 (3)	C15B—O8B	1.223 (16)
C15—C16	1.535 (3)	C15B—C16B	1.519 (16)
O8—Na1	2.298 (3)	O8B—Na1B	2.280 (17)
C16—Cl1	1.768 (3)	C16B—Cl1B	1.777 (16)
C16—H16A	0.9900	C16B—H16C	0.9900
C16—H16B	0.9900	C16B—H16D	0.9900
O9—C17	1.248 (4)	O9B—C17B	1.273 (19)
C17—N3	1.328 (3)	O9B—Mn1B^i^	2.258 (13)
C17—H17	0.9500	C17B—N3B	1.335 (17)
N3—C19	1.452 (4)	C17B—H17B	0.9500
N3—C18	1.458 (4)	N3B—C18B	1.434 (17)
C18—H18A	0.9800	N3B—C19B	1.466 (18)
C18—H18B	0.9800	C18B—H18D	0.9800
C18—H18C	0.9800	C18B—H18E	0.9800
C19—H19A	0.9800	C18B—H18F	0.9800
C19—H19B	0.9800	C19B—H19D	0.9800
C19—H19C	0.9800	C19B—H19E	0.9800
O10—C20	1.233 (3)	C19B—H19F	0.9800
O10—Mn1^i^	2.4178 (19)	O10B—C20B	1.243 (17)
O10—Na1	2.421 (3)	O10B—Na1B	2.95 (3)
C20—N4	1.319 (4)	C20B—N4B	1.336 (17)
C20—H20	0.9500	C20B—H20B	0.9500
N4—C21	1.449 (5)	N4B—C21B	1.44 (2)
N4—C22	1.472 (4)	N4B—C22B	1.481 (18)
C21—H21A	0.9800	C21B—H21D	0.9800
C21—H21B	0.9800	C21B—H21E	0.9800
C21—H21C	0.9800	C21B—H21F	0.9800
C22—H22A	0.9800	C22B—H22D	0.9800
C22—H22B	0.9800	C22B—H22E	0.9800
C22—H22C	0.9800	C22B—H22F	0.9800
O11—C23	1.237 (3)	O11B—C23B	1.270 (17)
O11—Na1	2.365 (2)	O11B—Na1B	2.386 (15)
C23—N5	1.328 (3)	C23B—N5B	1.278 (16)
C23—H23	0.9500	C23B—H23B	0.9500
N5—C25	1.452 (3)	N5B—C25B	1.413 (16)
N5—C24	1.462 (4)	N5B—C24B	1.477 (17)
C24—H24A	0.9800	C24B—H24D	0.9800
C24—H24B	0.9800	C24B—H24E	0.9800
C24—H24C	0.9800	C24B—H24F	0.9800
C25—H25A	0.9800	C25B—H25D	0.9800
C25—H25B	0.9800	C25B—H25E	0.9800
C25—H25C	0.9800	C25B—H25F	0.9800
O12—C26	1.172 (9)	O12B—C26B	1.193 (11)
C26—N6	1.308 (14)	C26B—N6B	1.333 (18)
C26—H26	0.9500	C26B—H26B	0.9500
N6—C28	1.409 (7)	N6B—C28B	1.439 (13)
N6—C27	1.453 (12)	N6B—C27B	1.44 (2)
C27—H27A	0.9800	C27B—H27D	0.9800
C27—H27B	0.9800	C27B—H27E	0.9800
C27—H27C	0.9800	C27B—H27F	0.9800
C28—H28A	0.9800	C28B—H28D	0.9800
C28—H28B	0.9800	C28B—H28E	0.9800
C28—H28C	0.9800	C28B—H28F	0.9800
Na1—O4^i^	2.494 (3)	Na1B—O1B^i^	2.32 (2)
Na1—O1^i^	2.547 (3)	Na1B—O4B^i^	2.34 (2)
Na1—Na1^i^	3.254 (4)	Na1B—Na1B^i^	3.04 (3)
Na1—Mn1^i^	3.488 (3)	Na1B—Mn2B^i^	3.51 (2)
Na1—Mn2^i^	3.577 (3)	Na1B—Mn1B^i^	3.68 (2)
Mn1B—O6B^i^	1.832 (10)		
			
O6^i^—Mn1—O1	177.26 (6)	O2B—Mn1B—O9B^i^	89.6 (5)
O6^i^—Mn1—O2	99.77 (6)	O6B^i^—Mn1B—O10B	92.7 (7)
O1—Mn1—O2	81.69 (5)	O1B—Mn1B—O10B	82.6 (7)
O6^i^—Mn1—N2^i^	89.69 (7)	N2B^i^—Mn1B—O10B	92.3 (7)
O1—Mn1—N2^i^	88.54 (6)	O2B—Mn1B—O10B	85.3 (6)
O2—Mn1—N2^i^	167.43 (7)	O9B^i^—Mn1B—O10B	173.1 (6)
O6^i^—Mn1—O9	89.47 (6)	O6B^i^—Mn1B—Na1B	134.1 (4)
O1—Mn1—O9	92.73 (5)	O1B—Mn1B—Na1B	41.8 (4)
O2—Mn1—O9	94.38 (5)	N2B^i^—Mn1B—Na1B	61.1 (5)
N2^i^—Mn1—O9	93.96 (7)	O2B—Mn1B—Na1B	109.6 (4)
O6^i^—Mn1—O10^i^	96.37 (6)	O9B^i^—Mn1B—Na1B	121.7 (5)
O1—Mn1—O10^i^	81.41 (6)	O10B—Mn1B—Na1B	56.2 (6)
O2—Mn1—O10^i^	85.64 (5)	O6B^i^—Mn1B—Na1B^i^	150.0 (4)
N2^i^—Mn1—O10^i^	85.09 (7)	O1B—Mn1B—Na1B^i^	32.2 (5)
O9—Mn1—O10^i^	174.07 (6)	N2B^i^—Mn1B—Na1B^i^	64.4 (4)
O6^i^—Mn1—Na1^i^	131.88 (6)	O2B—Mn1B—Na1B^i^	106.4 (4)
O1—Mn1—Na1^i^	45.40 (6)	O9B^i^—Mn1B—Na1B^i^	72.1 (5)
O2—Mn1—Na1^i^	101.88 (5)	O10B—Mn1B—Na1B^i^	104.8 (6)
N2^i^—Mn1—Na1^i^	65.56 (6)	Na1B—Mn1B—Na1B^i^	49.9 (5)
O9—Mn1—Na1^i^	130.54 (5)	N1B—O1B—Mn1B	113.1 (7)
O10^i^—Mn1—Na1^i^	43.91 (6)	N1B—O1B—Na1B^i^	116.1 (9)
N1—O1—Mn1	113.44 (10)	Mn1B—O1B—Na1B^i^	122.3 (7)
N1—O1—Na1	123.14 (11)	N1B—O1B—Na1B	112.8 (10)
Mn1—O1—Na1	121.24 (8)	Mn1B—O1B—Na1B	107.8 (7)
N1—O1—Na1^i^	101.75 (10)	Na1B^i^—O1B—Na1B	78.9 (7)
Mn1—O1—Na1^i^	102.84 (8)	C1B—N1B—O1B	114.1 (9)
Na1—O1—Na1^i^	81.06 (8)	C1B—N1B—Mn2B	130.8 (9)
C1—N1—O1	113.17 (15)	O1B—N1B—Mn2B	115.1 (8)
C1—N1—Mn2	131.64 (13)	C1B—O2B—Mn1B	111.1 (7)
O1—N1—Mn2	115.10 (11)	O2B—C1B—N1B	119.4 (10)
C1—O2—Mn1	111.27 (11)	O2B—C1B—C2B	122.2 (10)
O2—C1—N1	119.77 (16)	N1B—C1B—C2B	118.4 (10)
O2—C1—C2	119.92 (16)	C7B—C2B—C3B	120.6 (10)
N1—C1—C2	120.31 (16)	C7B—C2B—C1B	125.9 (11)
C3—C2—C7	119.72 (16)	C3B—C2B—C1B	113.4 (11)
C3—C2—C1	117.76 (18)	C4B—C3B—C2B	120.8 (12)
C7—C2—C1	122.53 (17)	C4B—C3B—H3B	119.6
C4—C3—C2	121.85 (18)	C2B—C3B—H3B	119.6
C4—C3—H3	119.1	C5B—C4B—C3B	119.0 (13)
C2—C3—H3	119.1	C5B—C4B—H4B	120.5
C3—C4—C5	118.04 (18)	C3B—C4B—H4B	120.5
C3—C4—H4	121.0	C4B—C5B—C6B	120.5 (13)
C5—C4—H4	121.0	C4B—C5B—H5B	119.8
C6—C5—C4	121.04 (18)	C6B—C5B—H5B	119.8
C6—C5—H5	119.5	C5B—C6B—C7B	121.2 (12)
C4—C5—H5	119.5	C5B—C6B—H6B	119.4
C5—C6—C7	121.36 (19)	C7B—C6B—H6B	119.4
C5—C6—H6	119.3	O3B—C7B—C2B	122.8 (11)
C7—C6—H6	119.3	O3B—C7B—C6B	119.5 (12)
O3—C7—C6	117.35 (18)	C2B—C7B—C6B	117.7 (10)
O3—C7—C2	124.71 (17)	C7B—O3B—Mn2B	130.4 (9)
C6—C7—C2	117.93 (16)	O3B—Mn2B—O4B	171.7 (5)
C7—O3—Mn2	131.17 (13)	O3B—Mn2B—O5B	98.9 (4)
O3—Mn2—O4	167.89 (6)	O4B—Mn2B—O5B	82.2 (3)
O3—Mn2—O5	97.09 (6)	O3B—Mn2B—N1B	88.9 (5)
O4—Mn2—O5	81.92 (5)	O4B—Mn2B—N1B	88.4 (4)
O3—Mn2—N1	89.50 (6)	O5B—Mn2B—N1B	165.2 (5)
O4—Mn2—N1	88.75 (6)	O3B—Mn2B—O7B	95.2 (6)
O5—Mn2—N1	164.73 (7)	O4B—Mn2B—O7B	93.0 (5)
O3—Mn2—O7	95.33 (6)	O5B—Mn2B—O7B	91.7 (5)
O4—Mn2—O7	96.77 (6)	N1B—Mn2B—O7B	100.2 (7)
O5—Mn2—O7	93.37 (6)	O3B—Mn2B—Na1B^i^	134.1 (5)
N1—Mn2—O7	99.75 (7)	O4B—Mn2B—Na1B^i^	38.2 (4)
O3—Mn2—Na1^i^	128.65 (6)	O5B—Mn2B—Na1B^i^	101.7 (5)
O4—Mn2—Na1^i^	41.22 (5)	N1B—Mn2B—Na1B^i^	64.2 (5)
O5—Mn2—Na1^i^	104.30 (6)	O7B—Mn2B—Na1B^i^	124.4 (4)
N1—Mn2—Na1^i^	61.24 (6)	O3B—Mn2B—Na1B	146.5 (5)
O7—Mn2—Na1^i^	128.56 (5)	O4B—Mn2B—Na1B	35.7 (4)
O3—Mn2—Na1	152.09 (6)	O5B—Mn2B—Na1B	113.1 (4)
O4—Mn2—Na1	34.32 (5)	N1B—Mn2B—Na1B	62.4 (5)
O5—Mn2—Na1	109.54 (6)	O7B—Mn2B—Na1B	75.0 (5)
N1—Mn2—Na1	66.86 (6)	Na1B^i^—Mn2B—Na1B	50.0 (5)
O7—Mn2—Na1	75.39 (6)	N2B—O4B—Mn2B	112.5 (7)
Na1^i^—Mn2—Na1	53.21 (6)	N2B—O4B—Na1B^i^	109.6 (10)
N2—O4—Mn2	113.41 (10)	Mn2B—O4B—Na1B^i^	112.0 (6)
N2—O4—Na1	114.44 (12)	N2B—O4B—Na1B	120.9 (9)
Mn2—O4—Na1	119.36 (9)	Mn2B—O4B—Na1B	117.1 (6)
N2—O4—Na1^i^	113.10 (12)	Na1B^i^—O4B—Na1B	79.6 (7)
Mn2—O4—Na1^i^	109.07 (8)	C8B—N2B—O4B	115.0 (10)
Na1—O4—Na1^i^	83.64 (8)	C8B—N2B—Mn1B^i^	129.5 (9)
C8—N2—O4	113.77 (14)	O4B—N2B—Mn1B^i^	115.1 (8)
C8—N2—Mn1^i^	130.21 (13)	C8B—O5B—Mn2B	112.2 (7)
O4—N2—Mn1^i^	116.02 (11)	O5B—C8B—N2B	118.0 (9)
C8—O5—Mn2	111.26 (11)	O5B—C8B—C9B	123.0 (10)
N2—C8—O5	119.30 (16)	N2B—C8B—C9B	119.0 (10)
N2—C8—C9	119.78 (17)	C14B—C9B—C10B	119.7 (11)
O5—C8—C9	120.87 (16)	C14B—C9B—C8B	126.8 (11)
C10—C9—C14	119.49 (17)	C10B—C9B—C8B	113.5 (11)
C10—C9—C8	117.61 (18)	C11B—C10B—C9B	120.1 (12)
C14—C9—C8	122.75 (17)	C11B—C10B—H10B	120.0
C11—C10—C9	121.92 (19)	C9B—C10B—H10B	120.0
C11—C10—H10	119.0	C10B—C11B—C12B	120.3 (14)
C9—C10—H10	119.0	C10B—C11B—H11B	119.8
C10—C11—C12	118.38 (18)	C12B—C11B—H11B	119.8
C10—C11—H11	120.8	C11B—C12B—C13B	119.6 (14)
C12—C11—H11	120.8	C11B—C12B—H12B	120.2
C13—C12—C11	120.62 (18)	C13B—C12B—H12B	120.2
C13—C12—H12	119.7	C14B—C13B—C12B	121.1 (13)
C11—C12—H12	119.7	C14B—C13B—H13B	119.4
C12—C13—C14	121.54 (19)	C12B—C13B—H13B	119.4
C12—C13—H13	119.2	C13B—C14B—O6B	119.2 (11)
C14—C13—H13	119.2	C13B—C14B—C9B	119.2 (11)
O6—C14—C13	117.32 (18)	O6B—C14B—C9B	121.5 (11)
O6—C14—C9	124.57 (17)	C14B—O6B—Mn1B^i^	131.5 (8)
C13—C14—C9	118.05 (16)	C15B—O7B—Mn2B	130.0 (11)
C14—O6—Mn1^i^	127.12 (12)	O8B—C15B—O7B	130.3 (17)
C15—O7—Mn2	130.06 (13)	O8B—C15B—C16B	116.2 (15)
O8—C15—O7	128.3 (2)	O7B—C15B—C16B	113.4 (14)
O8—C15—C16	120.6 (2)	C15B—O8B—Na1B	136.3 (15)
O7—C15—C16	111.06 (19)	C15B—C16B—Cl1B	119.7 (14)
C15—O8—Na1	135.95 (19)	C15B—C16B—H16C	107.4
C15—C16—Cl1	114.45 (18)	Cl1B—C16B—H16C	107.4
C15—C16—H16A	108.6	C15B—C16B—H16D	107.4
Cl1—C16—H16A	108.6	Cl1B—C16B—H16D	107.4
C15—C16—H16B	108.6	H16C—C16B—H16D	106.9
Cl1—C16—H16B	108.6	C17B—O9B—Mn1B^i^	117.4 (16)
H16A—C16—H16B	107.6	O9B—C17B—N3B	130 (3)
C17—O9—Mn1	122.96 (17)	O9B—C17B—H17B	115.2
O9—C17—N3	125.2 (3)	N3B—C17B—H17B	115.2
O9—C17—H17	117.4	C17B—N3B—C18B	127 (2)
N3—C17—H17	117.4	C17B—N3B—C19B	118 (2)
C17—N3—C19	121.6 (3)	C18B—N3B—C19B	115 (2)
C17—N3—C18	120.6 (3)	N3B—C18B—H18D	109.5
C19—N3—C18	117.7 (2)	N3B—C18B—H18E	109.5
N3—C18—H18A	109.5	H18D—C18B—H18E	109.5
N3—C18—H18B	109.5	N3B—C18B—H18F	109.5
H18A—C18—H18B	109.5	H18D—C18B—H18F	109.5
N3—C18—H18C	109.5	H18E—C18B—H18F	109.5
H18A—C18—H18C	109.5	N3B—C19B—H19D	109.5
H18B—C18—H18C	109.5	N3B—C19B—H19E	109.5
N3—C19—H19A	109.5	H19D—C19B—H19E	109.5
N3—C19—H19B	109.5	N3B—C19B—H19F	109.5
H19A—C19—H19B	109.5	H19D—C19B—H19F	109.5
N3—C19—H19C	109.5	H19E—C19B—H19F	109.5
H19A—C19—H19C	109.5	C20B—O10B—Mn1B	118.0 (15)
H19B—C19—H19C	109.5	C20B—O10B—Na1B	149.2 (19)
C20—O10—Mn1^i^	144.64 (16)	Mn1B—O10B—Na1B	82.5 (7)
C20—O10—Na1	118.91 (16)	O10B—C20B—N4B	128 (2)
Mn1^i^—O10—Na1	92.24 (8)	O10B—C20B—H20B	115.8
O10—C20—N4	125.0 (2)	N4B—C20B—H20B	115.8
O10—C20—H20	117.5	C20B—N4B—C21B	122 (2)
N4—C20—H20	117.5	C20B—N4B—C22B	120 (2)
C20—N4—C21	121.9 (2)	C21B—N4B—C22B	118 (2)
C20—N4—C22	121.0 (3)	N4B—C21B—H21D	109.5
C21—N4—C22	117.1 (3)	N4B—C21B—H21E	109.5
N4—C21—H21A	109.5	H21D—C21B—H21E	109.5
N4—C21—H21B	109.5	N4B—C21B—H21F	109.5
H21A—C21—H21B	109.5	H21D—C21B—H21F	109.5
N4—C21—H21C	109.5	H21E—C21B—H21F	109.5
H21A—C21—H21C	109.5	N4B—C22B—H22D	109.5
H21B—C21—H21C	109.5	N4B—C22B—H22E	109.5
N4—C22—H22A	109.5	H22D—C22B—H22E	109.5
N4—C22—H22B	109.5	N4B—C22B—H22F	109.5
H22A—C22—H22B	109.5	H22D—C22B—H22F	109.5
N4—C22—H22C	109.5	H22E—C22B—H22F	109.5
H22A—C22—H22C	109.5	C23B—O11B—Na1B	169.6 (19)
H22B—C22—H22C	109.5	O11B—C23B—N5B	122 (2)
C23—O11—Na1	146.1 (2)	O11B—C23B—H23B	118.8
O11—C23—N5	125.3 (3)	N5B—C23B—H23B	118.8
O11—C23—H23	117.4	C23B—N5B—C25B	136 (2)
N5—C23—H23	117.4	C23B—N5B—C24B	112.0 (17)
C23—N5—C25	122.4 (3)	C25B—N5B—C24B	111.5 (19)
C23—N5—C24	121.8 (2)	N5B—C24B—H24D	109.5
C25—N5—C24	115.7 (3)	N5B—C24B—H24E	109.5
N5—C24—H24A	109.5	H24D—C24B—H24E	109.5
N5—C24—H24B	109.5	N5B—C24B—H24F	109.5
H24A—C24—H24B	109.5	H24D—C24B—H24F	109.5
N5—C24—H24C	109.5	H24E—C24B—H24F	109.5
H24A—C24—H24C	109.5	N5B—C25B—H25D	109.5
H24B—C24—H24C	109.5	N5B—C25B—H25E	109.5
N5—C25—H25A	109.5	H25D—C25B—H25E	109.5
N5—C25—H25B	109.5	N5B—C25B—H25F	109.5
H25A—C25—H25B	109.5	H25D—C25B—H25F	109.5
N5—C25—H25C	109.5	H25E—C25B—H25F	109.5
H25A—C25—H25C	109.5	O12B—C26B—N6B	128.2 (13)
H25B—C25—H25C	109.5	O12B—C26B—H26B	115.9
O12—C26—N6	132.1 (10)	N6B—C26B—H26B	115.9
O12—C26—H26	113.9	C26B—N6B—C28B	131.7 (19)
N6—C26—H26	113.9	C26B—N6B—C27B	120.3 (11)
C26—N6—C28	126.6 (9)	C28B—N6B—C27B	107.6 (17)
C26—N6—C27	117.7 (7)	N6B—C27B—H27D	109.5
C28—N6—C27	115.7 (9)	N6B—C27B—H27E	109.5
N6—C27—H27A	109.5	H27D—C27B—H27E	109.5
N6—C27—H27B	109.5	N6B—C27B—H27F	109.5
H27A—C27—H27B	109.5	H27D—C27B—H27F	109.5
N6—C27—H27C	109.5	H27E—C27B—H27F	109.5
H27A—C27—H27C	109.5	N6B—C28B—H28D	109.5
H27B—C27—H27C	109.5	N6B—C28B—H28E	109.5
N6—C28—H28A	109.5	H28D—C28B—H28E	109.5
N6—C28—H28B	109.5	N6B—C28B—H28F	109.5
H28A—C28—H28B	109.5	H28D—C28B—H28F	109.5
N6—C28—H28C	109.5	H28E—C28B—H28F	109.5
H28A—C28—H28C	109.5	O8B—Na1B—O1B^i^	124.7 (12)
H28B—C28—H28C	109.5	O8B—Na1B—O4B^i^	165.7 (13)
O8—Na1—O11	107.89 (12)	O1B^i^—Na1B—O4B^i^	68.3 (7)
O8—Na1—O4	86.13 (10)	O8B—Na1B—O11B	108.9 (9)
O11—Na1—O4	165.43 (14)	O1B^i^—Na1B—O11B	85.6 (9)
O8—Na1—O10	87.15 (10)	O4B^i^—Na1B—O11B	76.1 (7)
O11—Na1—O10	92.11 (10)	O8B—Na1B—O4B	81.9 (8)
O4—Na1—O10	84.48 (9)	O1B^i^—Na1B—O4B	65.9 (6)
O8—Na1—O1	87.21 (11)	O4B^i^—Na1B—O4B	100.4 (7)
O11—Na1—O1	117.93 (12)	O11B—Na1B—O4B	150.0 (12)
O4—Na1—O1	65.44 (6)	O8B—Na1B—O1B	104.2 (10)
O10—Na1—O1	149.69 (10)	O1B^i^—Na1B—O1B	101.1 (7)
O8—Na1—O4^i^	146.95 (14)	O4B^i^—Na1B—O1B	64.9 (6)
O11—Na1—O4^i^	74.18 (9)	O11B—Na1B—O1B	133.8 (10)
O4—Na1—O4^i^	96.35 (8)	O4B—Na1B—O1B	65.0 (4)
O10—Na1—O4^i^	125.89 (12)	O8B—Na1B—O10B	79.2 (10)
O1—Na1—O4^i^	64.52 (7)	O1B^i^—Na1B—O10B	154.9 (10)
O8—Na1—O1^i^	143.57 (12)	O4B^i^—Na1B—O10B	87.2 (8)
O11—Na1—O1^i^	100.78 (11)	O11B—Na1B—O10B	94.0 (9)
O4—Na1—O1^i^	64.75 (7)	O4B—Na1B—O10B	115.8 (8)
O10—Na1—O1^i^	69.52 (9)	O1B—Na1B—O10B	61.7 (7)
O1—Na1—O1^i^	98.94 (8)	O8B—Na1B—Na1B^i^	129.3 (10)
O4^i^—Na1—O1^i^	62.61 (7)	O1B^i^—Na1B—Na1B^i^	52.6 (7)
O8—Na1—Na1^i^	126.36 (13)	O4B^i^—Na1B—Na1B^i^	51.3 (6)
O11—Na1—Na1^i^	120.03 (14)	O11B—Na1B—Na1B^i^	120.1 (12)
O4—Na1—Na1^i^	49.60 (6)	O4B—Na1B—Na1B^i^	49.2 (5)
O10—Na1—Na1^i^	112.26 (13)	O1B—Na1B—Na1B^i^	48.5 (6)
O1—Na1—Na1^i^	50.63 (7)	O10B—Na1B—Na1B^i^	107.9 (11)
O4^i^—Na1—Na1^i^	46.75 (7)	O8B—Na1B—Mn2B^i^	160.6 (8)
O1^i^—Na1—Na1^i^	48.30 (7)	O1B^i^—Na1B—Mn2B^i^	54.8 (5)
O8—Na1—Mn1^i^	113.30 (10)	O4B^i^—Na1B—Mn2B^i^	29.8 (3)
O11—Na1—Mn1^i^	114.19 (10)	O11B—Na1B—Mn2B^i^	52.6 (6)
O4—Na1—Mn1^i^	54.53 (6)	O4B—Na1B—Mn2B^i^	111.4 (7)
O10—Na1—Mn1^i^	43.85 (6)	O1B—Na1B—Mn2B^i^	94.4 (6)
O1—Na1—Mn1^i^	113.17 (7)	O10B—Na1B—Mn2B^i^	105.7 (7)
O4^i^—Na1—Mn1^i^	94.29 (8)	Na1B^i^—Na1B—Mn2B^i^	67.8 (7)
O1^i^—Na1—Mn1^i^	31.77 (4)	O8B—Na1B—Mn1B	111.8 (10)
Na1^i^—Na1—Mn1^i^	68.41 (8)	O1B^i^—Na1B—Mn1B	114.8 (8)
O8—Na1—Mn2^i^	163.39 (10)	O4B^i^—Na1B—Mn1B	54.0 (5)
O11—Na1—Mn2^i^	57.00 (7)	O11B—Na1B—Mn1B	105.6 (7)
O4—Na1—Mn2^i^	109.53 (9)	O4B—Na1B—Mn1B	95.4 (5)
O10—Na1—Mn2^i^	99.53 (9)	O1B—Na1B—Mn1B	30.5 (4)
O1—Na1—Mn2^i^	94.21 (8)	O10B—Na1B—Mn1B	41.3 (4)
O4^i^—Na1—Mn2^i^	29.71 (4)	Na1B^i^—Na1B—Mn1B	67.8 (8)
O1^i^—Na1—Mn2^i^	52.53 (5)	Mn2B^i^—Na1B—Mn1B	81.8 (4)
Na1^i^—Na1—Mn2^i^	65.13 (8)	O8B—Na1B—Mn2B	67.9 (6)
Mn1^i^—Na1—Mn2^i^	81.35 (6)	O1B^i^—Na1B—Mn2B	92.7 (5)
O8—Na1—Mn2	66.03 (8)	O4B^i^—Na1B—Mn2B	108.1 (6)
O11—Na1—Mn2	165.96 (12)	O11B—Na1B—Mn2B	174.5 (9)
O4—Na1—Mn2	26.32 (4)	O4B—Na1B—Mn2B	27.1 (3)
O10—Na1—Mn2	100.00 (8)	O1B—Na1B—Mn2B	51.6 (3)
O1—Na1—Mn2	50.87 (4)	O10B—Na1B—Mn2B	89.8 (6)
O4^i^—Na1—Mn2	103.76 (7)	Na1B^i^—Na1B—Mn2B	62.2 (5)
O1^i^—Na1—Mn2	90.17 (6)	Mn2B^i^—Na1B—Mn2B	130.0 (5)
Na1^i^—Na1—Mn2	61.66 (6)	Mn1B—Na1B—Mn2B	79.9 (4)
Mn1^i^—Na1—Mn2	79.70 (4)	O8B—Na1B—Mn1B^i^	99.7 (9)
Mn2^i^—Na1—Mn2	126.79 (6)	O1B^i^—Na1B—Mn1B^i^	25.5 (4)
O6B^i^—Mn1B—O1B	175.2 (6)	O4B^i^—Na1B—Mn1B^i^	92.6 (7)
O6B^i^—Mn1B—N2B^i^	91.2 (4)	O11B—Na1B—Mn1B^i^	99.1 (8)
O1B—Mn1B—N2B^i^	87.9 (4)	O4B—Na1B—Mn1B^i^	50.9 (4)
O6B^i^—Mn1B—O2B	99.0 (4)	O1B—Na1B—Mn1B^i^	106.1 (6)
O1B—Mn1B—O2B	81.8 (4)	O10B—Na1B—Mn1B^i^	166.4 (6)
N2B^i^—Mn1B—O2B	169.7 (4)	Na1B^i^—Na1B—Mn1B^i^	62.3 (8)
O6B^i^—Mn1B—O9B^i^	92.7 (5)	Mn2B^i^—Na1B—Mn1B^i^	79.9 (5)
O1B—Mn1B—O9B^i^	92.0 (6)	Mn1B—Na1B—Mn1B^i^	130.1 (5)
N2B^i^—Mn1B—O9B^i^	91.9 (6)	Mn2B—Na1B—Mn1B^i^	77.4 (3)
			
O2—Mn1—O1—N1	6.79 (11)	O12—C26—N6—C27	2.2 (19)
N2^i^—Mn1—O1—N1	−165.28 (12)	N2B^i^—Mn1B—O1B—N1B	−172.2 (12)
O9—Mn1—O1—N1	100.83 (11)	O2B—Mn1B—O1B—N1B	6.7 (11)
O10^i^—Mn1—O1—N1	−80.02 (11)	O9B^i^—Mn1B—O1B—N1B	96.0 (11)
Na1^i^—Mn1—O1—N1	−109.06 (13)	O10B—Mn1B—O1B—N1B	−79.6 (12)
O2—Mn1—O1—Na1	−156.91 (10)	Na1B—Mn1B—O1B—N1B	−125.4 (14)
N2^i^—Mn1—O1—Na1	31.02 (11)	Na1B^i^—Mn1B—O1B—N1B	146.7 (17)
O9—Mn1—O1—Na1	−62.87 (10)	N2B^i^—Mn1B—O1B—Na1B^i^	41.1 (9)
O10^i^—Mn1—O1—Na1	116.28 (10)	O2B—Mn1B—O1B—Na1B^i^	−140.0 (9)
Na1^i^—Mn1—O1—Na1	87.24 (10)	O9B^i^—Mn1B—O1B—Na1B^i^	−50.7 (9)
O2—Mn1—O1—Na1^i^	115.85 (7)	O10B—Mn1B—O1B—Na1B^i^	133.7 (10)
N2^i^—Mn1—O1—Na1^i^	−56.22 (8)	Na1B—Mn1B—O1B—Na1B^i^	87.9 (9)
O9—Mn1—O1—Na1^i^	−150.11 (7)	N2B^i^—Mn1B—O1B—Na1B	−46.8 (8)
O10^i^—Mn1—O1—Na1^i^	29.04 (7)	O2B—Mn1B—O1B—Na1B	132.1 (8)
Mn1—O1—N1—C1	−5.53 (18)	O9B^i^—Mn1B—O1B—Na1B	−138.6 (7)
Na1—O1—N1—C1	157.82 (13)	O10B—Mn1B—O1B—Na1B	45.8 (8)
Na1^i^—O1—N1—C1	−115.26 (15)	Na1B^i^—Mn1B—O1B—Na1B	−87.9 (9)
Mn1—O1—N1—Mn2	171.55 (7)	Mn1B—O1B—N1B—C1B	−7.5 (18)
Na1—O1—N1—Mn2	−25.10 (17)	Na1B^i^—O1B—N1B—C1B	141.3 (13)
Na1^i^—O1—N1—Mn2	61.82 (12)	Na1B—O1B—N1B—C1B	−130.2 (13)
Mn1—O2—C1—N1	6.2 (2)	Mn1B—O1B—N1B—Mn2B	173.6 (7)
Mn1—O2—C1—C2	−174.07 (12)	Na1B^i^—O1B—N1B—Mn2B	−37.5 (16)
O1—N1—C1—O2	−0.7 (2)	Na1B—O1B—N1B—Mn2B	51.0 (14)
Mn2—N1—C1—O2	−177.10 (12)	Mn1B—O2B—C1B—N1B	2.3 (18)
O1—N1—C1—C2	179.60 (14)	Mn1B—O2B—C1B—C2B	179.6 (10)
Mn2—N1—C1—C2	3.1 (3)	O1B—N1B—C1B—O2B	3 (2)
O2—C1—C2—C3	1.3 (2)	Mn2B—N1B—C1B—O2B	−178.0 (12)
N1—C1—C2—C3	−178.93 (17)	O1B—N1B—C1B—C2B	−174.2 (13)
O2—C1—C2—C7	−178.96 (16)	Mn2B—N1B—C1B—C2B	4 (2)
N1—C1—C2—C7	0.8 (3)	O2B—C1B—C2B—C7B	−171.8 (14)
C7—C2—C3—C4	−0.1 (3)	N1B—C1B—C2B—C7B	6 (2)
C1—C2—C3—C4	179.67 (17)	O2B—C1B—C2B—C3B	5.7 (19)
C2—C3—C4—C5	1.6 (3)	N1B—C1B—C2B—C3B	−176.9 (14)
C3—C4—C5—C6	−0.7 (3)	C7B—C2B—C3B—C4B	−3 (2)
C4—C5—C6—C7	−1.6 (3)	C1B—C2B—C3B—C4B	178.9 (14)
C5—C6—C7—O3	−176.11 (18)	C2B—C3B—C4B—C5B	6 (2)
C5—C6—C7—C2	3.0 (3)	C3B—C4B—C5B—C6B	−5 (2)
C3—C2—C7—O3	176.88 (17)	C4B—C5B—C6B—C7B	1 (2)
C1—C2—C7—O3	−2.8 (3)	C3B—C2B—C7B—O3B	−177.8 (14)
C3—C2—C7—C6	−2.2 (3)	C1B—C2B—C7B—O3B	0 (2)
C1—C2—C7—C6	178.07 (16)	C3B—C2B—C7B—C6B	0 (2)
C6—C7—O3—Mn2	179.96 (14)	C1B—C2B—C7B—C6B	177.3 (13)
C2—C7—O3—Mn2	0.9 (3)	C5B—C6B—C7B—O3B	179.1 (14)
C7—O3—Mn2—O4	−79.8 (3)	C5B—C6B—C7B—C2B	1 (2)
C7—O3—Mn2—O5	−164.35 (17)	C2B—C7B—O3B—Mn2B	−16 (2)
C7—O3—Mn2—N1	1.83 (18)	C6B—C7B—O3B—Mn2B	166.3 (12)
C7—O3—Mn2—O7	101.57 (18)	C7B—O3B—Mn2B—O5B	−173.7 (14)
C7—O3—Mn2—Na1^i^	−49.6 (2)	C7B—O3B—Mn2B—N1B	19.0 (15)
C7—O3—Mn2—Na1	33.0 (3)	C7B—O3B—Mn2B—O7B	−81.2 (15)
O3—Mn2—O4—N2	−90.4 (3)	C7B—O3B—Mn2B—Na1B^i^	70.4 (17)
O5—Mn2—O4—N2	−4.27 (12)	C7B—O3B—Mn2B—Na1B	−11 (2)
N1—Mn2—O4—N2	−172.15 (12)	O5B—Mn2B—O4B—N2B	3.5 (9)
O7—Mn2—O4—N2	88.19 (12)	N1B—Mn2B—O4B—N2B	172.1 (10)
Na1^i^—Mn2—O4—N2	−127.01 (14)	O7B—Mn2B—O4B—N2B	−87.7 (10)
Na1—Mn2—O4—N2	139.54 (16)	Na1B^i^—Mn2B—O4B—N2B	124.0 (12)
O3—Mn2—O4—Na1	130.1 (3)	Na1B—Mn2B—O4B—N2B	−146.7 (12)
O5—Mn2—O4—Na1	−143.81 (10)	O5B—Mn2B—O4B—Na1B^i^	−120.4 (7)
N1—Mn2—O4—Na1	48.31 (10)	N1B—Mn2B—O4B—Na1B^i^	48.1 (8)
O7—Mn2—O4—Na1	−51.35 (10)	O7B—Mn2B—O4B—Na1B^i^	148.3 (8)
Na1^i^—Mn2—O4—Na1	93.45 (11)	Na1B—Mn2B—O4B—Na1B^i^	89.3 (8)
O3—Mn2—O4—Na1^i^	36.6 (3)	O5B—Mn2B—O4B—Na1B	150.3 (7)
O5—Mn2—O4—Na1^i^	122.74 (8)	N1B—Mn2B—O4B—Na1B	−41.2 (8)
N1—Mn2—O4—Na1^i^	−45.14 (8)	O7B—Mn2B—O4B—Na1B	59.0 (8)
O7—Mn2—O4—Na1^i^	−144.80 (8)	Na1B^i^—Mn2B—O4B—Na1B	−89.3 (8)
Na1—Mn2—O4—Na1^i^	−93.45 (11)	Mn2B—O4B—N2B—C8B	−4.2 (16)
Mn2—O4—N2—C8	2.5 (2)	Na1B^i^—O4B—N2B—C8B	121.1 (12)
Na1—O4—N2—C8	144.12 (15)	Na1B—O4B—N2B—C8B	−149.6 (11)
Na1^i^—O4—N2—C8	−122.34 (15)	Mn2B—O4B—N2B—Mn1B^i^	−178.2 (6)
Mn2—O4—N2—Mn1^i^	−178.17 (8)	Na1B^i^—O4B—N2B—Mn1B^i^	−52.9 (11)
Na1—O4—N2—Mn1^i^	−36.57 (17)	Na1B—O4B—N2B—Mn1B^i^	36.5 (14)
Na1^i^—O4—N2—Mn1^i^	56.96 (15)	Mn2B—O5B—C8B—N2B	0.7 (16)
O4—N2—C8—O5	2.3 (3)	Mn2B—O5B—C8B—C9B	−175.7 (10)
Mn1^i^—N2—C8—O5	−176.91 (14)	O4B—N2B—C8B—O5B	2 (2)
O4—N2—C8—C9	−175.10 (15)	Mn1B^i^—N2B—C8B—O5B	175.2 (11)
Mn1^i^—N2—C8—C9	5.7 (3)	O4B—N2B—C8B—C9B	178.8 (12)
Mn2—O5—C8—N2	−5.7 (2)	Mn1B^i^—N2B—C8B—C9B	−8 (2)
Mn2—O5—C8—C9	171.64 (13)	O5B—C8B—C9B—C14B	−175.5 (13)
N2—C8—C9—C10	−175.29 (18)	N2B—C8B—C9B—C14B	8 (2)
O5—C8—C9—C10	7.4 (3)	O5B—C8B—C9B—C10B	2 (2)
N2—C8—C9—C14	9.2 (3)	N2B—C8B—C9B—C10B	−174.7 (14)
O5—C8—C9—C14	−168.17 (17)	C14B—C9B—C10B—C11B	0 (2)
C14—C9—C10—C11	0.1 (3)	C8B—C9B—C10B—C11B	−177.0 (15)
C8—C9—C10—C11	−175.56 (18)	C9B—C10B—C11B—C12B	1 (3)
C9—C10—C11—C12	0.2 (3)	C10B—C11B—C12B—C13B	−2 (3)
C10—C11—C12—C13	−0.7 (3)	C11B—C12B—C13B—C14B	1 (3)
C11—C12—C13—C14	0.7 (3)	C12B—C13B—C14B—O6B	179.0 (14)
C12—C13—C14—O6	176.92 (17)	C12B—C13B—C14B—C9B	0 (2)
C12—C13—C14—C9	−0.3 (3)	C10B—C9B—C14B—C13B	−1 (2)
C10—C9—C14—O6	−177.13 (17)	C8B—C9B—C14B—C13B	176.4 (14)
C8—C9—C14—O6	−1.7 (3)	C10B—C9B—C14B—O6B	−179.8 (13)
C10—C9—C14—C13	−0.1 (3)	C8B—C9B—C14B—O6B	−3 (2)
C8—C9—C14—C13	175.39 (17)	C13B—C14B—O6B—Mn1B^i^	178.1 (11)
C13—C14—O6—Mn1^i^	161.57 (13)	C9B—C14B—O6B—Mn1B^i^	−3 (2)
C9—C14—O6—Mn1^i^	−21.4 (2)	Mn2B—O7B—C15B—O8B	−9 (5)
Mn2—O7—C15—O8	3.2 (3)	Mn2B—O7B—C15B—C16B	171.9 (17)
Mn2—O7—C15—C16	−178.50 (15)	O7B—C15B—O8B—Na1B	10 (6)
O7—C15—O8—Na1	−26.3 (4)	C16B—C15B—O8B—Na1B	−172 (2)
C16—C15—O8—Na1	155.5 (2)	O8B—C15B—C16B—Cl1B	−5 (4)
O8—C15—C16—Cl1	−13.9 (3)	O7B—C15B—C16B—Cl1B	174 (2)
O7—C15—C16—Cl1	167.63 (17)	Mn1B^i^—O9B—C17B—N3B	−173 (4)
Mn1—O9—C17—N3	178.7 (3)	O9B—C17B—N3B—C18B	16 (8)
O9—C17—N3—C19	178.0 (4)	O9B—C17B—N3B—C19B	−150 (5)
O9—C17—N3—C18	−5.4 (7)	Mn1B—O10B—C20B—N4B	175 (4)
Mn1^i^—O10—C20—N4	42.6 (6)	Na1B—O10B—C20B—N4B	−58 (6)
Na1—O10—C20—N4	−168.6 (5)	O10B—C20B—N4B—C21B	18 (9)
O10—C20—N4—C21	0.1 (9)	O10B—C20B—N4B—C22B	−159 (4)
O10—C20—N4—C22	−178.0 (4)	Na1B—O11B—C23B—N5B	42 (12)
Na1—O11—C23—N5	49.5 (5)	O11B—C23B—N5B—C25B	−171 (4)
O11—C23—N5—C25	174.8 (3)	O11B—C23B—N5B—C24B	−1 (4)
O11—C23—N5—C24	−0.6 (5)	O12B—C26B—N6B—C28B	178.1 (18)
O12—C26—N6—C28	−179.9 (10)	O12B—C26B—N6B—C27B	6 (3)

Symmetry code: (i) −*x*+1, −*y*, −*z*+1.

### Hydrogen-bond geometry (Å, º)

**Table:**

*D*—H···*A*	*D*—H	H···*A*	*D*···*A*	*D*—H···*A*
C18—H18*A*···O11^ii^	0.98	2.63	3.516 (5)	151
C19—H19*B*···O12	0.98	2.33	3.201 (5)	148
C20—H20···O8	0.95	2.64	3.279 (3)	125
C21—H21*B*···O2^i^	0.98	2.48	3.433 (3)	165
C24—H24*B*···O8	0.98	2.53	3.459 (4)	158
C25—H25*B*···O12^iii^	0.98	2.55	3.309 (6)	134
C25—H25*C*···O2^iii^	0.98	2.50	3.423 (5)	157
C26—H26···O7	0.95	2.49	3.339 (10)	149

Symmetry codes: (i) −*x*+1, −*y*, −*z*+1; (ii) −*x*+1/2, *y*+1/2, −*z*+1/2; (iii) −*x*+1/2, *y*−1/2, −*z*+1/2.
